# Large-Scale Discovery of Microbial Fibrillar Adhesins and Identification of Novel Members of Adhesive Domain Families

**DOI:** 10.1128/jb.00107-22

**Published:** 2022-05-24

**Authors:** Vivian Monzon, Alex Bateman

**Affiliations:** a European Molecular Biology Laboratory, European Bioinformatics Institute (EMBL-EBI), Hinxton, United Kingdom; Queen Mary University of London

**Keywords:** fibrillar adhesins, host-pathogen interaction, random forest classification, protein domain families, adhesive domains, structure prediction methods, AlphaFold2

## Abstract

Fibrillar adhesins are bacterial cell surface proteins that mediate interactions with the environment, including host cells during colonization or other bacteria during biofilm formation. These proteins are characterized by a stalk that projects the adhesive domain closer to the binding target. Fibrillar adhesins evolve quickly and thus can be difficult to computationally identify, yet they represent an important component for understanding bacterium-host interactions. To detect novel fibrillar adhesins, we developed a random forest prediction approach based on common characteristics we identified for this protein class. We applied this approach to *Firmicutes* and *Actinobacteria* proteomes, yielding over 6,500 confidently predicted fibrillar adhesins. To verify the approach, we investigated predicted fibrillar adhesins that lacked a known adhesive domain. Based on these proteins, we identified 24 sequence clusters representing potential novel members of adhesive domain families. We used AlphaFold to verify that 15 clusters showed structural similarity to known adhesive domains, such as the TED domain. Overall, our study has made a significant contribution to the number of known fibrillar adhesins and has enabled us to identify novel members of adhesive domain families involved in bacterial pathogenesis.

**IMPORTANCE** Fibrillar adhesins are a class of bacterial cell surface proteins that enable bacteria to interact with their environment. We developed a machine learning approach to identify fibrillar adhesins and applied this classification approach to the *Firmicutes* and *Actinobacteria* Reference Proteomes database. This method allowed us to detect a high number of novel fibrillar adhesins and also novel members of adhesive domain families. To confirm our predictions of these potential adhesin protein domains, we predicted their structure using the AlphaFold tool.

## INTRODUCTION

Fibrillar adhesins are an important class of bacterial surface proteins which are expressed by a wide range of bacterial species to mediate binding interactions. Essential binding targets include different host surface structures, such as extracellular matrix proteins. Pathogenic colonization and infection can occur as a consequence of the binding interactions to host cells (1). Single fibrillar adhesins have therefore been studied in depth and have been the focal point for antiadhesion therapies (2, 3). Fibrillar adhesins have also been described to mediate biofilm formation (4, 5).

Fibrillar adhesins are a recently defined class of proteins that led to a domain-based characterization and identification of these proteins across a wide range of bacterial species (6, 7). Key characteristics of fibrillar adhesins are their large length and an adhesion region, a rodlike repetitive region, and a cell surface anchor. The repetitive region contains repeating protein domains, also called stalk domains, which fold into a filamentous stalk. It has been suggested that the stalk projects the binding region closer to the binding target (8) and enables the adhesive region to be presented outside the cell by reaching beyond the surface layer. The repeats can vary in number, leading to fibrillar adhesins with stalks of different lengths. Proteins with varying repeat numbers of stalk domains between related bacterial strains have recently been termed “periscope proteins” (9). Whelan et al. propose that the varying length is used as a regulation mechanism to facilitate the binding to targets at different distances (9). The varying length may also lead to the adhesive region being differentially displayed beyond the surface layer based on the number of repeats. Several adhesive domains have been identified when studying the binding regions of bacterial adhesins, of which most bind to protein ligands, some bind to carbohydrates, and one adhesive domain is known to bind to ice crystals (10). Nevertheless, undoubtedly, a large number of adhesive domains remain undiscovered.

In our previous work, we have used the presence of known adhesive and stalk domains identified in fibrillar adhesins to detect more than 3,000 fibrillar adhesins-like (FA-like) proteins across all bacterial species based on profile hidden Markov model (HMM) searches (7). The limitation of this domain-based discovery approach is that only FA-like proteins with known adhesive and stalk domains are found. Not all adhesive and stalk domains are identified yet, and finding novel binding proteins or domains is important for the understanding of emerging bacterial interactions. To overcome this limitation, we studied the properties of FA-like proteins and developed a random forest-based discovery approach. The aim of this study is to enable the identification of FA-like proteins on a large scale, including those lacking a known adhesive or stalk domain. We apply our newly developed machine learning approach to the *Firmicutes* and *Actinobacteria* UniProt Reference Proteomes database and verify the approach by predicting the structure of *Firmicutes* FA-like proteins lacking a known adhesive domain with AlphaFold (11). Our approach facilitates the identification of relevant proteins during bacterial infection processes, enabling the investigation of novel members of adhesive domain families, leading to a better understanding of microbial interaction mechanisms.

## RESULTS

### Random forest classification.

The aim of this study is the extension of the identification of fibrillar adhesins compared to the domain-based approach described in our earlier study (7). To achieve this goal, additional identification features were combined with the presence of adhesive and stalk domains, although the adhesive and stalk domains clearly remain the most important features in this study. To undertake the identification approach based on the selected features, a random forest approach was selected. This is composed of individual decision trees classifying the proteins by sets of maximums of three features of all identification features provided as input to the algorithm and returning the strongest class as the prediction.

We decided to concentrate on the *Firmicutes* and *Actinobacteria* phyla in this study. Fibrillar adhesins are best studied in *Firmicutes*, and the cell surface composition and FA-like protein architecture of the *Actinobacteria* resemble those of the *Firmicutes* (3, 7). We created a positive training set based on the FA-like proteins identified in our previous study (7). For the negative training set, we randomly selected non-FA-like proteins from eight *Firmicutes* and *Actinobacteria* reference proteomes, in which FA-like proteins were detected in our previous study (7). Although an individual proteome may contain several FA-like proteins, they are present at relatively low numbers per proteome (0% to 1.47%), thus randomly selecting the negative examples is unlikely to select many, if any, true FA-like proteins (7). In total, the training set consists of 3,332 proteins, equally balanced between the positive and negative training sets. Using this training set, common properties for this protein class were determined.

**FIG 1:**
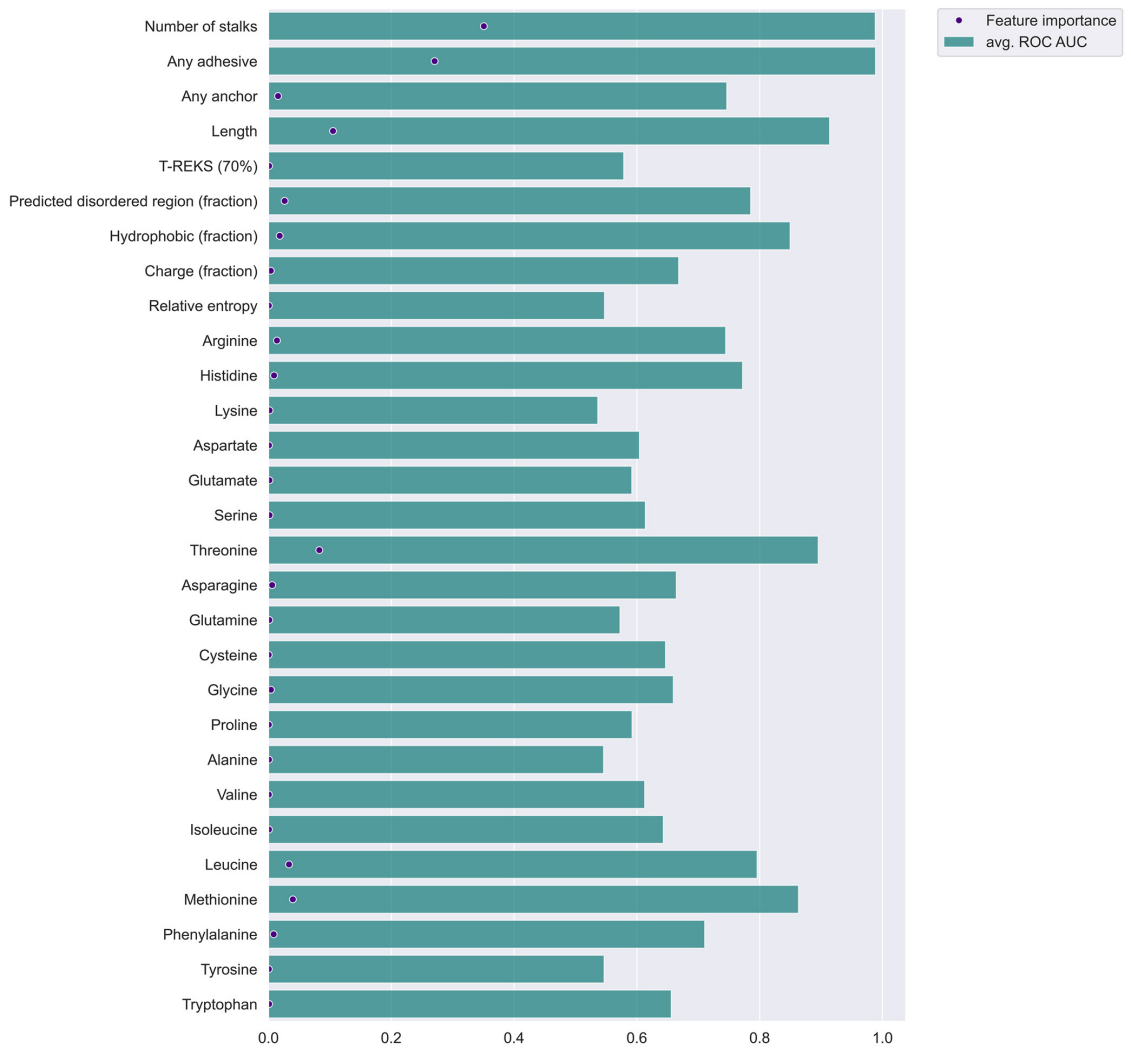
Bar plot showing the relative importance of prediction features. This plot visualizes the importance of each feature for the random forest classification. The bars show the calculated ROC AUC per feature when using it alone for classification. The dot represents the feature importance as calculated by the feature importance attribute implemented in the sklearn.ensemble random forest classifier.

Nearly all proteins of the positive training set (98%) have at least one adhesive and one stalk domain, whereas less than 0.1% of the proteins in the negative training set possess a known adhesive or stalk domain. Hence, the adhesive and stalk domains are the strongest identification features for this protein class (Fig. 1). To increase the chance of detecting proteins with unidentified stalk domains, we selected the presence of tandem sequence repeats with a minimum of 70% sequence identity allowed by the T-REKS tool (12). In periscope proteins, the stalk domains are described to be found in highly identical tandem repeats (9). But compared to periscope proteins, not all stalk domains in FA-like proteins are found in highly identical tandem repeats and are therefore missed by T-REKS. We observed that the stalk domain region tends to have a biased sequence composition and tends to be predicted as disordered despite known structures being found in some of these regions. We used the fraction of predicted disordered residues by IUPRED2 as an additional feature (13).

Fibrillar adhesins are attached to the bacterial cell surface. Known anchor domains or sortase motifs were found in 846 out of 1,666 proteins of the positive training set and were used as another identification feature.

FA-like proteins are among the longest proteins in the proteome; thus, the protein length turned out to be one of the strongest prediction features (Fig. 1). Their long length facilitates FA-like proteins to cross the peptidoglycan layer, which can be around 20 to 50 nm wide, depending on the bacterial species (14). The average protein length of the positive training set is 1,196 residues compared to 300 residues for the negative training set. Hence, for a longer sequence length of a protein, the probability increases that the protein functions as an FA-like protein.

To characterize the protein sequence of FA-like proteins, the amount of charged, as well as hydrophobic, amino acids per protein was selected as an additional feature. The protein sequences of the positive training data tend to have a slightly lower fraction of charged amino acids and tend to have a lower fraction of hydrophobic amino acids than the negative training data set.

Finally, we selected features related to the sequence composition. We calculated the fraction of each amino acid per sequence as well as the relative entropy describing the sequence composition bias. We observed that the relative entropy tends to be slightly higher in the positive training set and that threonine is 1.8-fold increased and leucine is 1.5-fold decreased in the positive training set compared to the negative training set (see Fig. S1 in the supplemental material).

**FIG 2:**
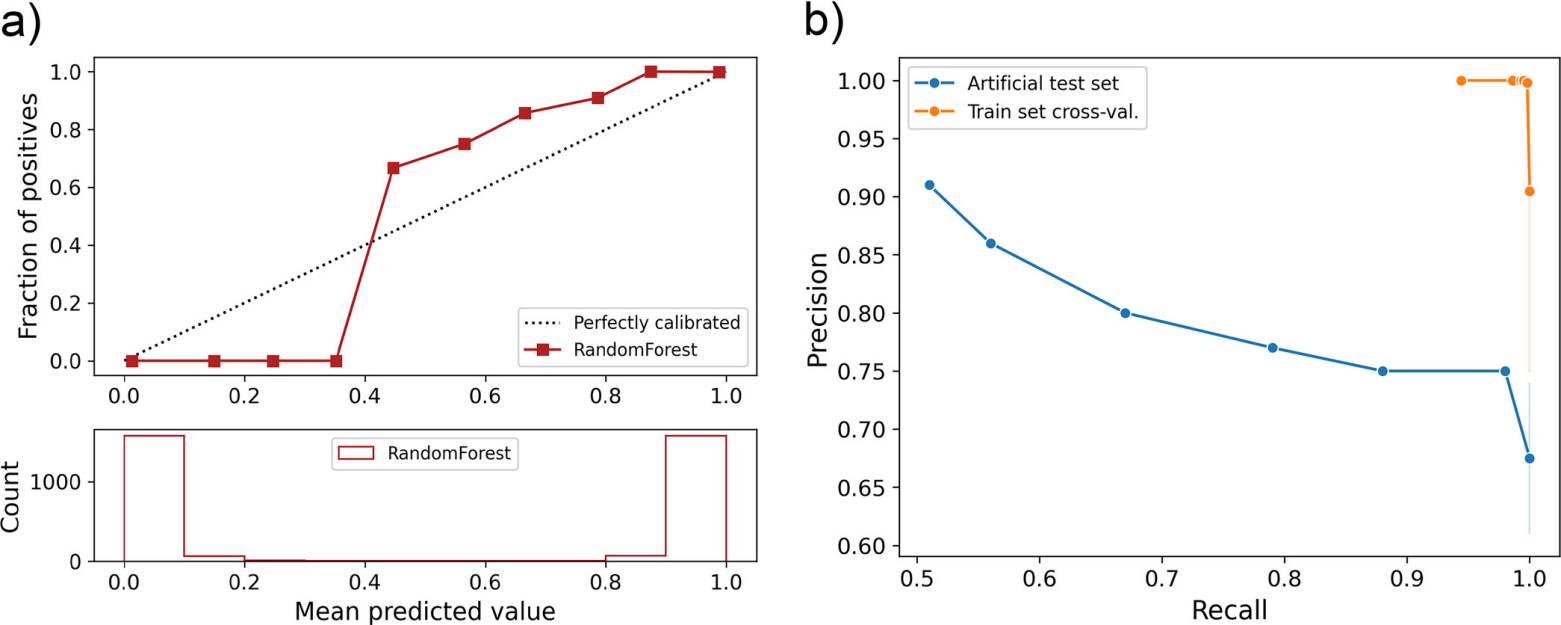
Validation of the trained random forest classifier. (a, Top) Subplot represents the reliability curve showing the observed fraction of predicted proteins belonging to the positive training data set against the expected fraction of positives. (a, Bottom) Subplot indicates the total number of proteins of the training set predicted per prediction score. (b) Precision recall curves calculated with the training set by using a cross-validation approach (orange) or using a test set with a positive set of FA-like proteins with adhesive and stalk domains artificially removed (blue).

We implemented the selected features in a random forest classification approach and analyzed the feature importance in the classification prediction based on the training data (Fig. 1). The adhesive and stalk domain features can yield a receiver operating characteristic (ROC) area under the curve (AUC) of 0.99 due to the fact that the training set is built upon FA-like proteins identified in our previous work using known adhesive and stalk domains (Fig. 1). The low diversity of the training data, considering that the positive training data nearly solely consist of proteins with at least one adhesive and one stalk domain, is reflected in the reliability and precision recall curve (Fig. 2a and b). The reliability curve shows the high number of proteins of the negative training set predicted with a prediction score below 0.1 and the high number of proteins of the positive training set predicted with a score above 0.9. Even though the number of predicted proteins with a score between 0.2 and 0.8 is low, the ratio of false positives tends to increase in the prediction score ranges of 0.5 to 0.7. To test if the training set leads to an overfitted model, we evaluated the random forest classification approach with an extreme case of FA-like proteins that we artificially present to have no adhesive or stalk domains (all other features of the proteins are retained), yielding a precision score of 0.8 and achieving a recall score of 0.67 due to missed true matches (Fig. 2b).

An important challenge of this work is to determine the random forest score threshold that will reliably identify novel FA-like proteins that potentially lack known stalk or adhesive domains.

### Analysis of predicted FA-like proteins.

When applying the classification approach to the *Firmicutes* and *Actinobacteria* UniProt reference proteomes, 45,444 FA-like proteins with a prediction score above 0.5 were identified, 24,197 proteins in *Firmicutes* and 21,247 proteins in *Actinobacteria* (Table 1). These represent 0.49% and 0.32% of the total number of reference proteins, respectively. The reference proteomes with the highest fraction of predicted FA-like proteins with a score of 0.7 or above are listed in Table S1. Here, we provide an analysis to help to determine a reasonable threshold to apply for downstream analysis and application of the classifier in general.

**TABLE 1:** Number of FA-like proteins discovered in *Firmicutes* and *Actinobacteria* per prediction score bin

Phylum	No. of FA-like proteins per score of:					
	0.5–0.6	0.6–0.7	0.7–0.8	0.8–0.9	0.9–1.0	1.0
*Firmicutes*	6,325	7,110	5,751	2,694	1,130	1,187
*Actinobacteria*	6,715	8,718	4,121	924	303	466

**FIG 3:**
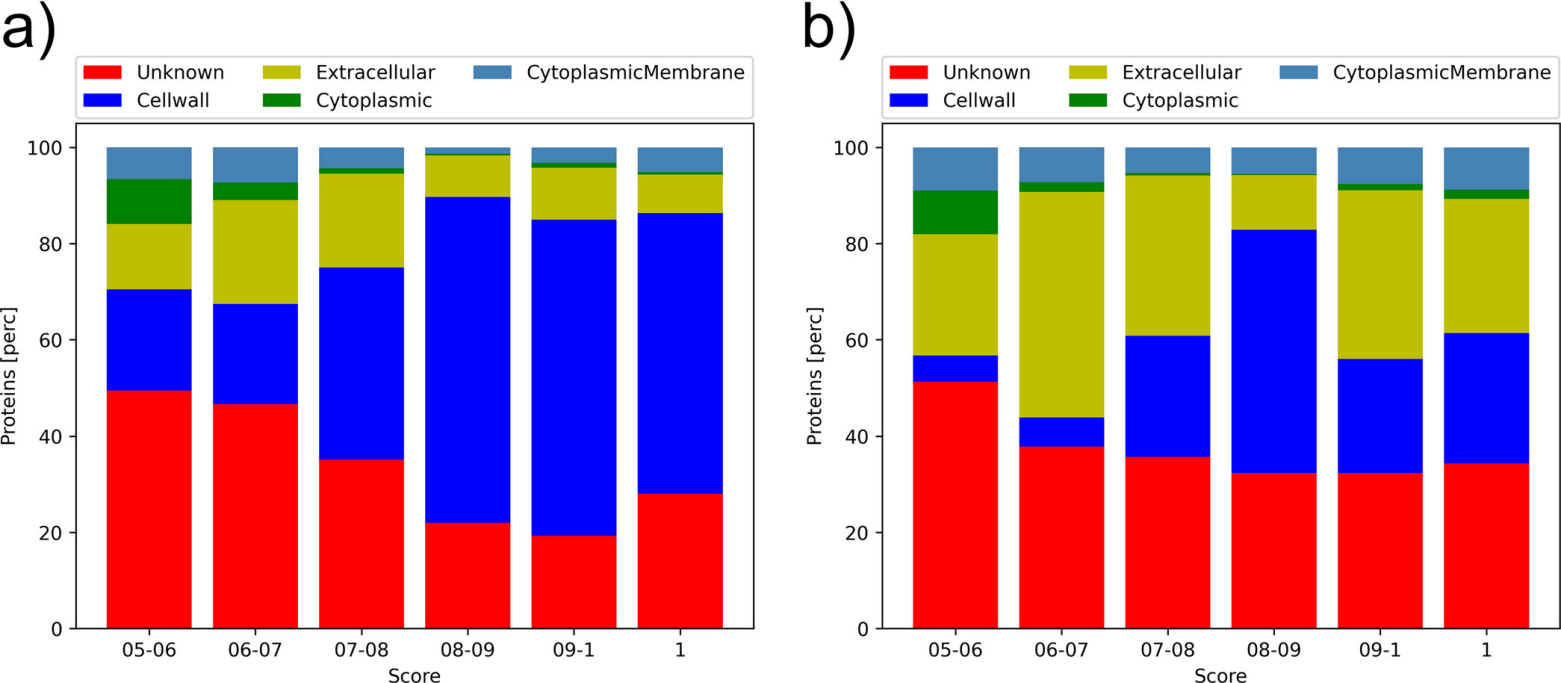
Predicted subcellular localization by PSORTb-predicted subcellular localizations for FA-like proteins predicted with a prediction score between 0.5 and 1.0 for *Firmicutes* (a) and *Actinobacteria* (b).

To study the characteristics of the predicted FA-like proteins (at a threshold > 0.5), we predicted their subcellular localizations using PSORTb (version 3) (15). For nearly half of the predicted FA-like proteins in *Firmicutes* with a prediction score of 0.7 or below, no localization was predicted, whereas the majority of FA-like proteins with a prediction score above 0.7 are predicted to be localized at the cell wall (Fig. 3a). For most of the predicted FA-like proteins in *Actinobacteria*, no localization was predicted by PSORTb. The highest protein numbers with a predicted localization are said to be extracellular, with around half of the FA-like proteins with a prediction score between 0.8 and 0.9 being predicted to be localized at the cell wall (Fig. 3b). Consequently, the cell wall anchor characteristic of FA-like proteins is more strongly represented in the predicted proteins in *Firmicutes* than *Actinobacteria*.

Even though most of the proteins (>75%) predicted with a score of 1.0 with known stalk domains have an adhesive domain, over 90% of the predicted FA-like proteins above a prediction score of 0.5 with known stalk domains have no known adhesive domain (Table 2 and 3). Given that our training set is biased toward FA-like proteins with both stalk and adhesive domains, this result may suggest that there exists a large number of FA-like proteins with as-yet-undiscovered or unannotated adhesive domains.

**TABLE 2:** Overview of the presence and absence of known adhesive and stalk domains for *Firmicutes*^*a*^

Domain	No. of FA-like proteins per score of:					
	0.5–0.6	0.6–0.7	0.7–0.8	0.8–0.9	0.9–1.0	1.0
Stalk solely	4,254	6,023	5,322	2,574	854	179
Adhesive solely	423	281	121	58	26	3
Adhesive and stalk	0	1	4	3	246	1,003
Neither adhesive nor stalk	1,648	805	304	59	4	2

a The number of proteins per prediction score category was counted, differentiating between proteins with an adhesive and/or stalk domains or neither an adhesive nor a stalk domain.

**TABLE 3:** Overview of the presence and absence of known adhesive and stalk domains for *Actinobacteria*^*a*^

Domain	No. of FA-like proteins per score of:					
	0.5–0.6	0.6–0.7	0.7–0.8	0.8–0.9	0.9–1.0	1.0
Stalk solely	4,782	7,472	3,588	808	211	171
Adhesive solely	468	527	370	73	5	6
Adhesive and stalk	0	2	3	0	80	288
Neither adhesive nor stalk	1,465	717	160	43	7	1

a The number of proteins per prediction score category was counted, differentiating between proteins with an adhesive and/or stalk domains or neither an adhesive nor a stalk domain.

We investigated the predicted proteins missing an adhesive domain and found adhesive domain-like sequences (Table 4). These were searched with the known adhesive domains using HMMER with a higher (less significant) E-value threshold of 1.0. Already, over 60% of the proteins with a prediction score of 1.0 have a known adhesive domain (Table 4). Adding the number of proteins with distantly related adhesive domains leads to an increase up to 6.72% for a prediction score between 0.8 to 0.9 in *Firmicutes* (Table 4). These results suggest the presence of novel adhesive domain families distantly related to existing ones detected in the predicted FA-like proteins. The results also indicate the possible existence of potential novel adhesive domains, unrelated to known adhesive domains.

**TABLE 4:** Distantly related adhesive domains^*a*^

Domain	% (no.) of total predicted FA-like proteins per score of:					
	0.5–0.6	0.6–0.7	0.7–0.8	0.8–0.9	0.9–1.0	1.0
*Firmicutes* (only known adhesive domains)	6.69 (423)	3.97 (282)	2.17 (125)	2.26 (61)	24.07 (272)	84.75 (1,006)
*Firmicutes* (with distantly related adhesive domains)	9.98 (631)	8.28 (589)	7.55 (434)	8.98 (242)	28.76 (325)	84.92 (1,008)
*Actinobacteria* (only known adhesive domains)	6.97 (468)	6.07 (529)	0.9 (37)	7.9 (73)	28.05 (85)	63.09 (294)
*Actinobacteria* (with distantly related adhesive domains)	7.55 (507)	7.66 (668)	3.45 (142)	10.71 (99)	28.38 (86)	63.09 (294)

a Shown are the percentages of the total predicted FA-like proteins with only known adhesive domains and additionally with the distantly related adhesive domains found by using a less significant E-value.

Taking the observed results into account, we suggest a high confidence scoring threshold of 0.8 since the predicted FA-like proteins with a scoring threshold below 0.8 might include false positives. Nevertheless, a scoring threshold of 0.7 can be used to find an extended set of FA-like proteins as long as a careful verification of the predicted proteins is carried out.

### Verification of novel adhesive domains.

To verify the random forest-based discovery approach, we further investigated the predicted FA-like proteins in the *Firmicutes* Reference Proteomes database. These include proteins without known adhesive or stalk domains. Here, we are interested in the proteins with known stalk domains that lack a known adhesive domain. We observed that many of them have a domain annotation gap at the N-terminus, distal to the cell surface anchor. Of these proteins, we selected a subset of proteins with a minimum of 4 stalk domains. These were 1,546 proteins in *Firmicutes* with a prediction score above 0.5. Under the assumption that these annotation gaps might include an adhesive region of the proteins, we clustered the N-terminal sequences into homologous sequence clusters using BLASTp (16). We selected the clusters with more than 5 sequences and with an average prediction score of 0.7 or above for further investigation, resulting in 24 clusters.

To further investigate the clusters, we chose one representative sequence per cluster (Table S2). We carried out two analyses, (i) to search for overlapping known Pfam domains, using the highly sensitive iterative Jackhmmer search, and (ii) to predict the structure of the sequence using AlphaFold (11, 17). Using Jackhmmer can be considered a more sensitive version of our previous analysis where we used a less significant HMMER threshold for known Pfam adhesive domains. Using Jackhmmer, we were able to detect even more distant similarities to known domains. To find out more about the function, particularly for sequences without overlapping Pfam domains, we searched with the predicted AlphaFold structure models against the PDB database for similar structures (18) (Table S3). We aligned the sequences of each cluster and built profile HMMs specific to each cluster. To understand the relative abundance of each of our clusters, we then searched for homologous sequences in UniProtKB and UniProt Reference Proteomes databases, as well as in the metagenomic MGnify database (19, 20).

### Clusters with sequence similarities to known adhesive domains.

**FIG 4:**
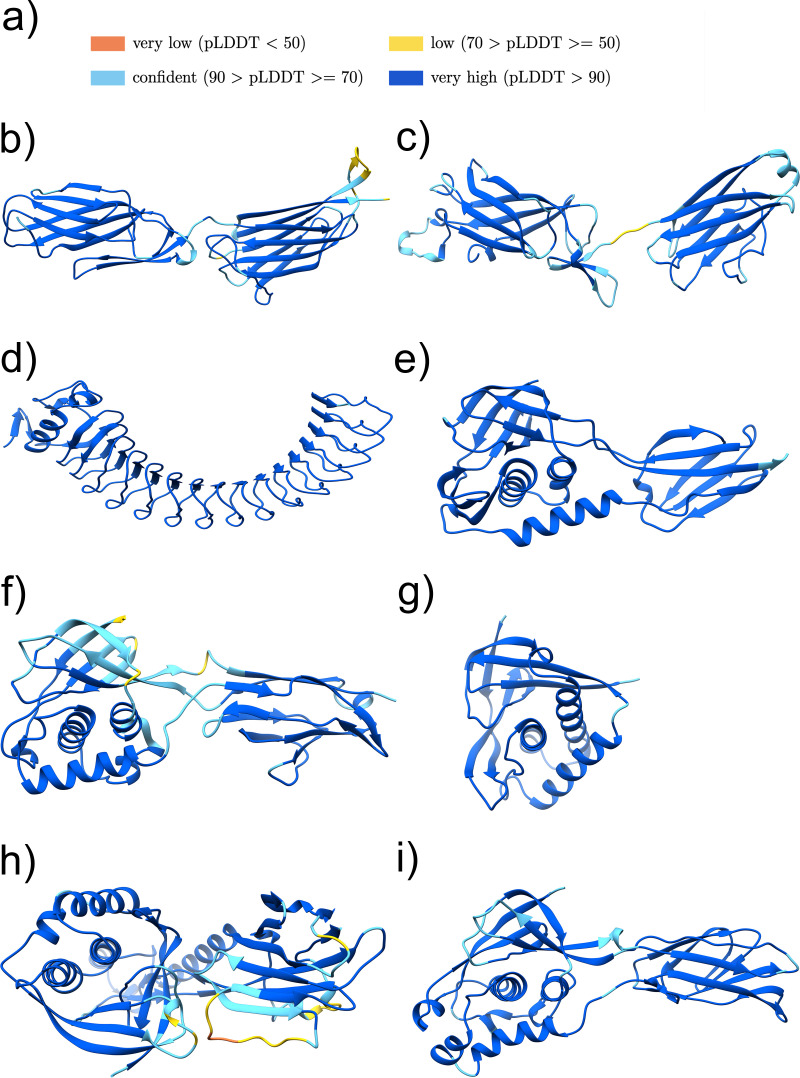
Structure models of cluster groups with sequence-similar adhesive domains found by Jackhmmer. (a) Color legend representing the quality of the AlphaFold models. (b to i) Structure models of the potential adhesive domain of cluster 2 (UniProtKB accession no. KF8EVB1 pos. 117-417) (b), cluster 6 (UniProtKB accession no. V2XMF4 pos. 80-366) (c), cluster 15 (UniProtKB accession no. A0A4U7JL97 pos. 37-420) (d), cluster 8 (UniProtKB accession no. R3TX93 pos. 40-276) (e), cluster 17 (UniProtKB accession no. A0A2Z4U801 pos. 240-508) (f), cluster 19 (UniProtKB accession no. S0JHJ4 pos. 32-199) (g), cluster 21 (UniProtKB accession no. A0A373LEL7 pos. 30-420) (h), and cluster 24 (UniProtKB accession no. B1BZ86 pos. 36-324) (i). The figures were produced using Chimera (42).

The Jackhmmer search using the putative adhesive region indicated 8 out of the 24 sequence clusters (cluster numbers 2, 6, 8, 15, 17, 19, 21, and 24) to be similar to known adhesive domain sequences (Table 5). These sequence similarities were confirmed by the DALI search with the AlphaFold predicted structure models (Fig. 4a to i; Fig. S2a to i).

**TABLE 5:** Information about clusters with detectable sequence similarities to known Pfam adhesive domains

Cluster no.	Avg. score^*a*^	Cluster size (no. of sequences)	Domain overlap^*b*^	UniProtKB^*c*^	UniProt Reference Proteomes^*c*^	MGnify^*c*^
2	0.93 ± 0.06	8	Big_8 (PF17961), Collagen_bind (PF05737)	487^*d*^	19^*d*^	650^*d*^
6	0.86 ± 0.05	6	Collagen_bind (PF05737)	276^*d*^	66^*d*^	1,174^*d*^
8	0.84 ± 0.09	28	TED (PF08341)	1,149	222	48,395
15	0.78 ± 0.11	8	LRR_4 (PF12799)	45,022	2,793	170,274
17	0.78 ± 0.05	47	TED (PF08341)	1,170	170	54,096
19	0.77 ± 0.07	39	TED (PF08341)	954	148	28,283
21	0.74 ± 0.03	7	TED (PF08341)	56^*e*^	16^*e*^	3,617^*e*^
24	0.71 ± 0.05	13	TED (PF08341)	1,223	214	53,312

a Data shown are the average protein prediction score per cluster.

b Similar Pfam adhesive domains found with Jackhmmer are indicated.

c The numbers of homologous sequence hits per cluster in the UniProtKB, UniProt Reference Proteomes, and MGnify databases are shown.

d C-terminal domain.

e N-terminal domain.

**(i) Cluster 2.** The predicted structure of cluster 2 representative sequence (UniProtKB accession no. K8EVB1) shows that it contains two distinct domains (Fig. 4b). For the N-terminal domain, Jackhmmer identified a Big_8 domain (Pfam ID PF17961), which is found in a variety of bacterial adhesins such as the Staphylococcus aureus proteins FnBPA, ClfA, and ClfB. A DALI search with the N-terminal domain strengthens the Jackhmmer results, as the best hit is the N-terminal Big_8 domain of the FnBPA binding region (PDB ID 4B60:B). For the C-terminal end of the C-terminal domain of cluster 2, the Jackhmmer search indicates an overlap with the Collagen_bind domain (Pfam ID PF05737). The second domain structure yields, with the DALI search, the Streptococcus gordonii adhesin Sgo0707, where the structure superposes to the Sgo0707_N2 domain (Pfam ID PF20623; PDB ID 4IGB:B). The subsequent DALI hits are the SdrG_C_C adhesive domain (Pfam ID PF10425; PDB ID 4JDZ:A) (Fig. S2b). The SdrG_C_C domain is also often found associated with the Big_8 domain in known adhesins and is likely to be homologous to the Collagen_bind domain. Cluster group 2 includes the Enterococcus faecalis protein with the gene name *ef2505* or *fss2*, which was described to bind to fibrinogen and play an important role in the adherence and virulence of E. faecalis (21). Thus, we propose that proteins in cluster 2 are likely to bind fibrinogen or other animal extracellular matrix proteins.

**(ii) Cluster 6.** For cluster 6, AlphaFold predicted a structure composed of two domains (Fig. 4c). The DALI search indicates, similar to cluster 2, for the N-terminal domain, a Big_8 domain as the top hit (PDB ID 5CF3:A), and for the C-terminal domain, a Collagen_bind domain as the best match (PDB ID 2Z1P:A) (Fig. S2c), thereby confirming the Jackhmmer search results. The Big_8 domain functions together with the Collagen_bind domain as a supradomain, enabling the binding to collagen by the collagen hug binding mechanism (22).

**(iii) Cluster 15.** Cluster group 15 includes the Listeria monocytogenes InternalinJ (UniProtKB accession no. Q8Y3L4), for which the crystal structure of the adhesive domain exists (PDB 3BZ5:A) (23), which was found using DALI with the predicted AlphaFold structure, reflecting the high accuracy of AlphaFold (Fig. 4d; Fig. S2d). The adhesive domain is formed by a series of leucine-rich repeats that are not matched by the LRR_4 family (Pfam ID PF12799) in Pfam. Proteins related to this class are one of the most prevalent that we found, with over 170,000 homologues identified in the MGnify protein sequence database.

**(iv) Cluster 8.** The Jackhmmer search, as well as the DALI search, with the AlphaFold prediction indicate cluster 8 being a class II TED (Pfam ID PF08341) adhesive domain (PDB ID 6FX6:A) (Fig. 4e; Fig. S2e), whose binding partner is unknown (24, 25). The TED adhesive domain is categorized into a class I and class II TED domain, depending on an additional N-terminal indel forming an alpha helix or an additional C-terminal indel folding into a beta-sandwich, respectively (24). Cluster group 8 includes the fibrinogen binding E. faecalis Fss3 protein (UniProtKB accession no. Q833P7), as well as an E. faecalis protein (UniProtKB accession no. Q831Z5) encoded by the virulence-associated ef2347 gene (21).

**(v) Cluster 17.** The Jackhmmer search indicates cluster 17 to also be distantly related to the TED adhesive domain (25). The DALI results, with the best hit being a class II TED domain (PDB ID 6FWV) (Fig. 4f; Fig. S2f), strengthen this hypothesis. To confirm that cluster 17 is a class II TED domain, we extended the representative sequence above the 400-residue limit by around 100 residues in order to include the characteristic C-terminal indel.

**(vi) Cluster 19.** Again, a TED domain was indicated by the Jackhmmer search for cluster 19 and supported by the DALI results (PDB ID 6FX6:A) (Fig. 4g; Fig. S2g). Analyzing the domain topology showed that there exists no seven-stranded beta-sandwich insertion, which is present in class II TED domains, whereby two beta-strands (A′ and B′) missing in class I TED domains are present (24). An existing or missing seven-stranded beta-sandwich insertion is the main criteria of the TED classification, suggesting that cluster 19 is a potential class I TED domain.

**(vii) Cluster 21.** The predicted structure of cluster 21 showed two distinct domains (Fig. 4h; Fig. S2h). The Jackhmmer search again indicated a TED domain. This is supported for the N-terminal domain by the DALI results, which indicated a class II TED domain. As in cluster 19, the seven-stranded beta-sandwich insertion is not present, but the two beta-strands, A′ and B′, are. The C-terminal domain structure includes 10 beta-strands and two longer alpha helices. The best DALI hit for the C-terminal domain is a stalk-like structure (PDB ID 3KPT:A), which only aligns with the beta-strands of the domain. Interestingly, the N-terminal TED-like domain is predicted to interact with the C-terminal domain.

**(viii) Cluster 24.** The Jackhmmer search, as well as the DALI results, indicates a TED domain for cluster 24 (PDB ID 6FWY:B). The existing beta-stranded insertion clearly characterizes this TED domain as a class II TED domain (Fig. 4i and Fig. S2i).

The sequences of five of the eight above-described clusters show similarity to known TED adhesive domains. TED domains were previously described to show a high sequence diversity (25).

### Clusters with structure models indicate a role in adhesion function.

The cluster groups 1, 3, 4, 5, 10, 12, and 13 do not yield persuasive Jackhmmer matches (Table 6), but their predicted structures resemble structures of known adhesive domains or adhesion-associated domains, suggesting that these clusters might be novel domains with potential binding functions.

**TABLE 6:** Investigation of the potential novel adhesive domains

Cluster no.	Avg. score^*a*^	Cluster size (no. of sequences)	Domain overlap^*b*^	UniProtKB^*c*^	UniProt Reference Proteomes^*c*^	MGnify^*c*^
1	0.93 ± 0.09	7		256	38	569
3	0.91 ± 0.05	9		502	122	1,124
4	0.89 ± 0.06	8		145	32	157
5	0.87 ± 0.06	11	MBG (PF17883) (C-terminal)	53	24	125
10	0.82 ± 0.07	10		2,359	551	6,668
12	0.82 ± 0.05	6	Cthe_2159 (PF14262)	495	65	1,495
13	0.8 ± 0.08	8		444	47	1,180

a Data shown are the average protein prediction score per cluster.

b Data show overlapping known Pfam domain families found with Jackhmmer.

c The numbers of homologous sequence hits per cluster in the UniProtKB, UniProt Reference Proteomes, and MGnify databases are shown.

### Clusters with jelly roll-resembling structure predictions.

**FIG 5:**
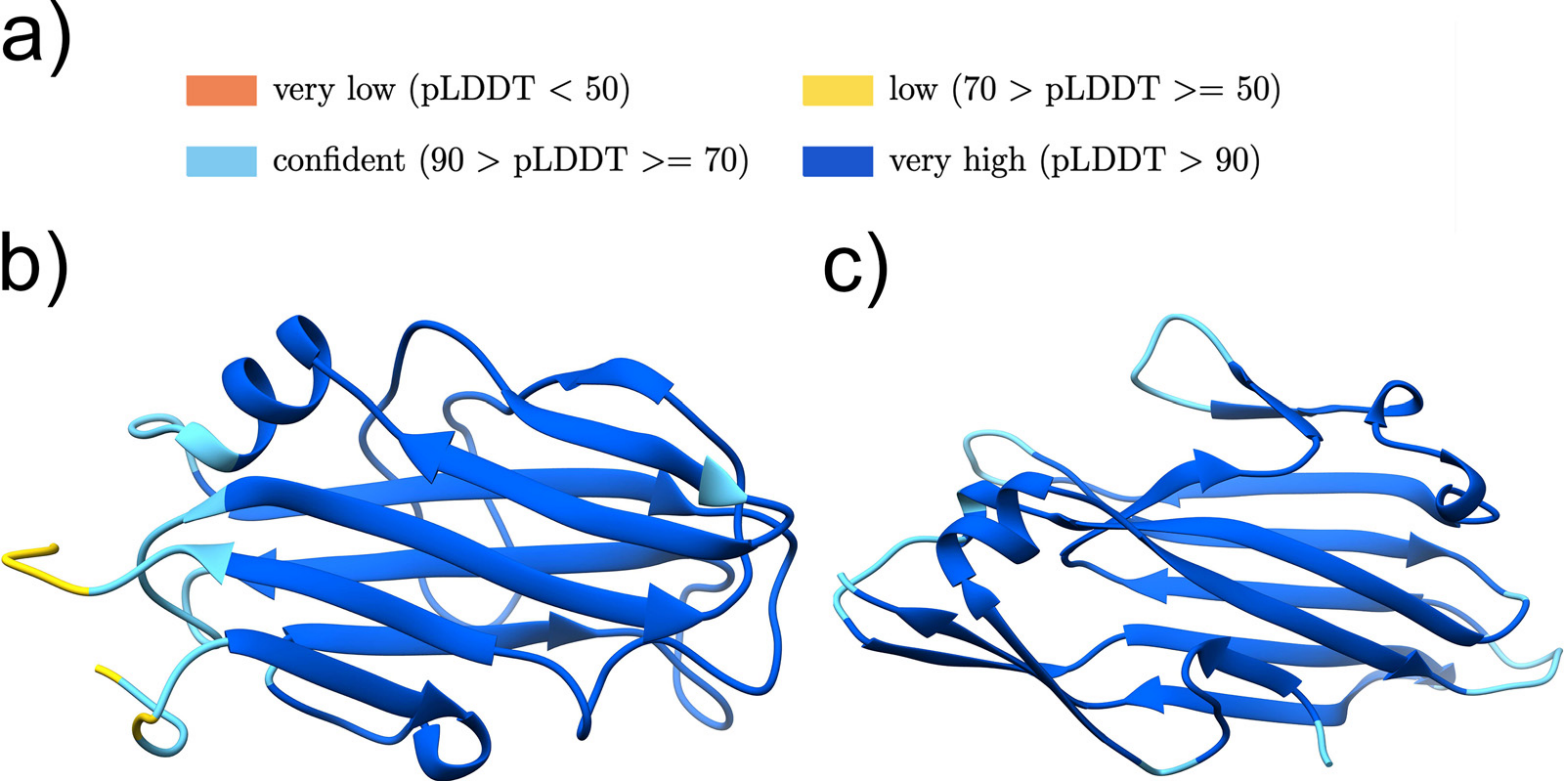
Potential novel adhesive domains with jelly roll-like predicted structures. (a) Color legend representing the quality of the AlphaFold models. (b and c) Structure models of the potential adhesive domain of cluster 4 (UniProtKB accession no. B0S3M8 pos. 153-316) (b) and cluster 5 (UniProtKB accession no. A0A5R8Q9T8 pos. 68-240) (c). The figures were produced using Chimera (42).

The structure predictions for clusters 4 and 5 are jelly roll like-structures (Fig. 5a to c).

The best DALI hit for cluster group 4 was a GramPos_pilinBB domain of the RrgB pilus backbone (PDB ID 2X9X:A) (Fig. 5b; Fig. S3b).

The Jackhmmer search for cluster group 5 showed an overlap with the MBG stalk domain at the C-terminus of the sequence, which was trimmed off, leaving a significant N-terminal sequence region for which the structure was predicted. The top DALI matches are related to surface adhesins, with the best match being a Clostridium perfringens pilin protein (PDB ID 5XCB:A) with the GramPos_pilinBB domain (Pfam ID PF16569) (Fig. S3c).

The Evolutionary Classification Of protein Domains (ECOD) databases classify the GramPos_pilinBB domain under the topology named “Common fold of diphtheria toxin/transcription factors/cytochrome f” (26). This category also includes the adhesive domains SdrG_C_C and Collagen_bind. The Collagen_bind adhesive domain is a well-studied jelly roll structure (27), which is composed of two antiparallel beta-sheets and two short alpha helices. Both AlphaFold structures for clusters 4 and 5 fold into jelly roll-like structures and show a high similarity to the Collagen_bind domain (PDB ID 1AMX:A). The structure surfaces seem to provide a groove on the beta-sheets, indicating a potential collagen binding site (27). However, the Collagen_bind domain alone has a 10-fold-lower collagen binding affinity than the collagen hug binding mechanisms based on the Big_8:Collagen_bind supradomain (22). The similarity to the Collagen_bind structure, and also the N-terminal protein position distal to the cell surface anchor, strongly suggests an adhesive function for clusters 4 and 5.

### Beta-solenoid fold cluster structure models.

**FIG 6:**
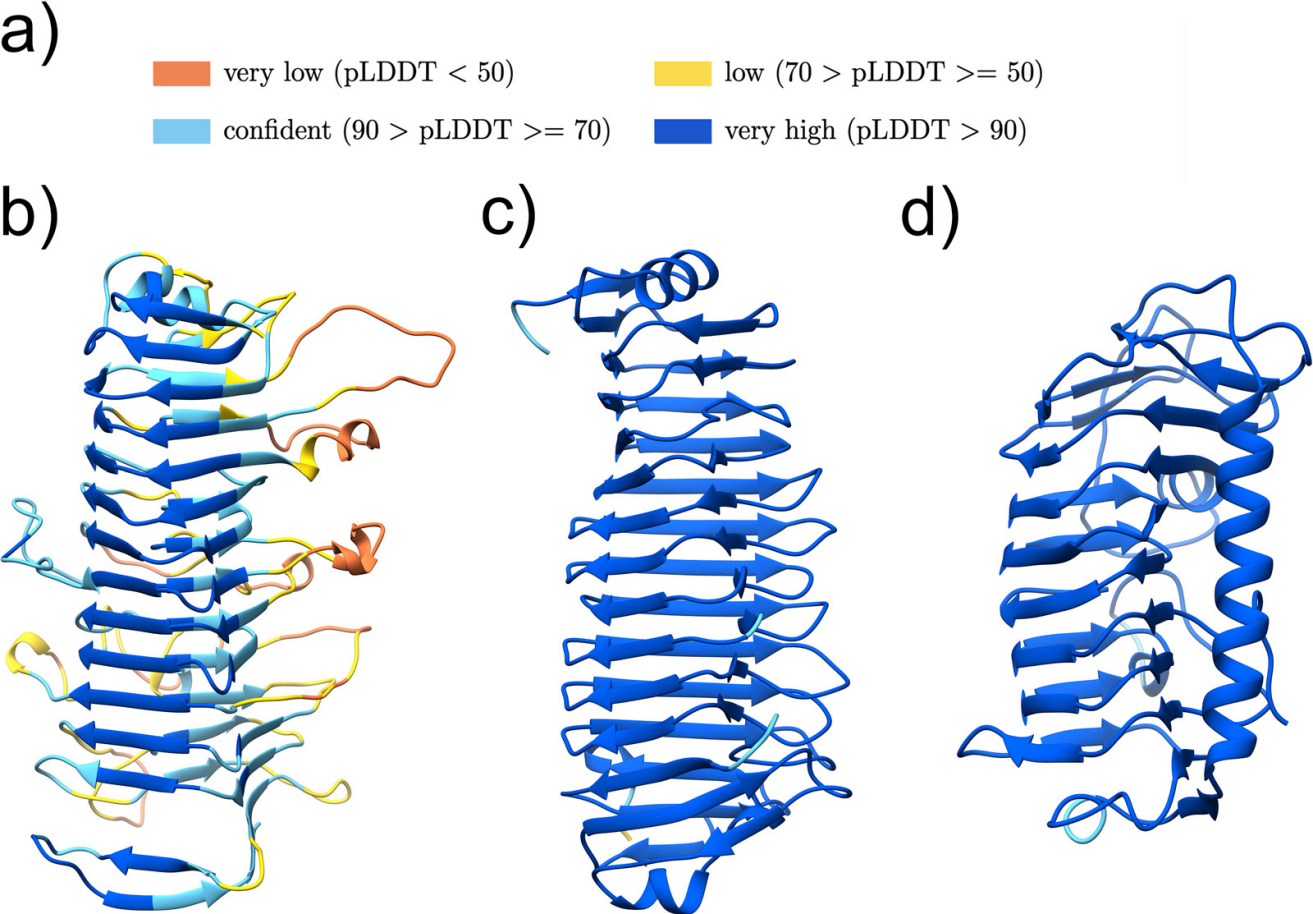
Predicted structures for clusters with potential novel adhesive domains, whose structures, but not sequences, seem to be related to known adhesive domains. (a) Color legend representing the quality of the AlphaFold models. Structure models of the potential adhesive domain of cluster 1 (UniProtKB accession no. A0A2Z6T9E9 pos. 185-718) (b), cluster 12 (UniProtKB accession no. A0A099WCN8 pos. 48-417) (c), and cluster 10 (UniProtKB accession no. A0A0F7RLJ7 pos. 46-326) (d) The figures were produced using Chimera (42).

Clusters 1, 10, and 12 are predicted to fold into beta-solenoid structures (Fig. 6a to d). The predicted structures for cluster groups 1 and 12 are most similar to the binding region of the serine-rich repeat protein (SRRP) from Lactobacillus reuteri strain 100-23C (PDB ID 5NY0:A) (Fig. S4b and c), being described to bind to epithelial cells and pectic acids and play a role in biofilm formation (28). The Jackhmmer search for cluster 12 already indicated, after the second iteration, a distant relation to the carbohydrate binding Cthe_2159 (Pfam ID PF14262) domain, which is part of the pectate lyase superfamily, whereas the DALI search clearly indicated the highest similarity to the L. reuteri SRRP adhesive region (28). The SRRP protein is not part of any existing Pfam family. Although we limited the clustering sequence to 400 residues, we investigated whether the domains were longer with AlphaFold and extended it in the case of cluster 1 to about 800 residues. Interestingly, cluster 1 is found on a Staphylococcus epidermidis protein with a SasG_G5-E stalk (UniProtKB accession no. A0A3G1RMM4), which was, so far, only found associated with the Bact_lectin adhesive domain (Pfam ID PF18483) in S. epidermidis and S. aureus SasG homologues (29). In our previous study, we discussed the possibility that an adhesive domain can function with any arbitrary stalk (7). The described example underlines this hypothesis and furthermore indicates a possible transfer of the adhesive domain onto a given stalk, increasing the adhesins variability. The SasG_G5-E stalk is also described to promote biofilm formation (29).

Cluster group 10 resembles an ice binding domain (PDB ID 4NUH:A) (Fig. S4d), where the representative protein sequence (UniProtKB accession no. A0A0F7RLJ7) is identical to a Bacillus anthracis protein (UniProtKB accession no. A0A384LNE7) with the gene name *BA_0871* or *BASH2_04951*, which was described to be collagen binding and to be linked to bacterial pathogenicity (30).

### Remaining clusters with structure models indicating potential adhesion function.

**FIG 7:**
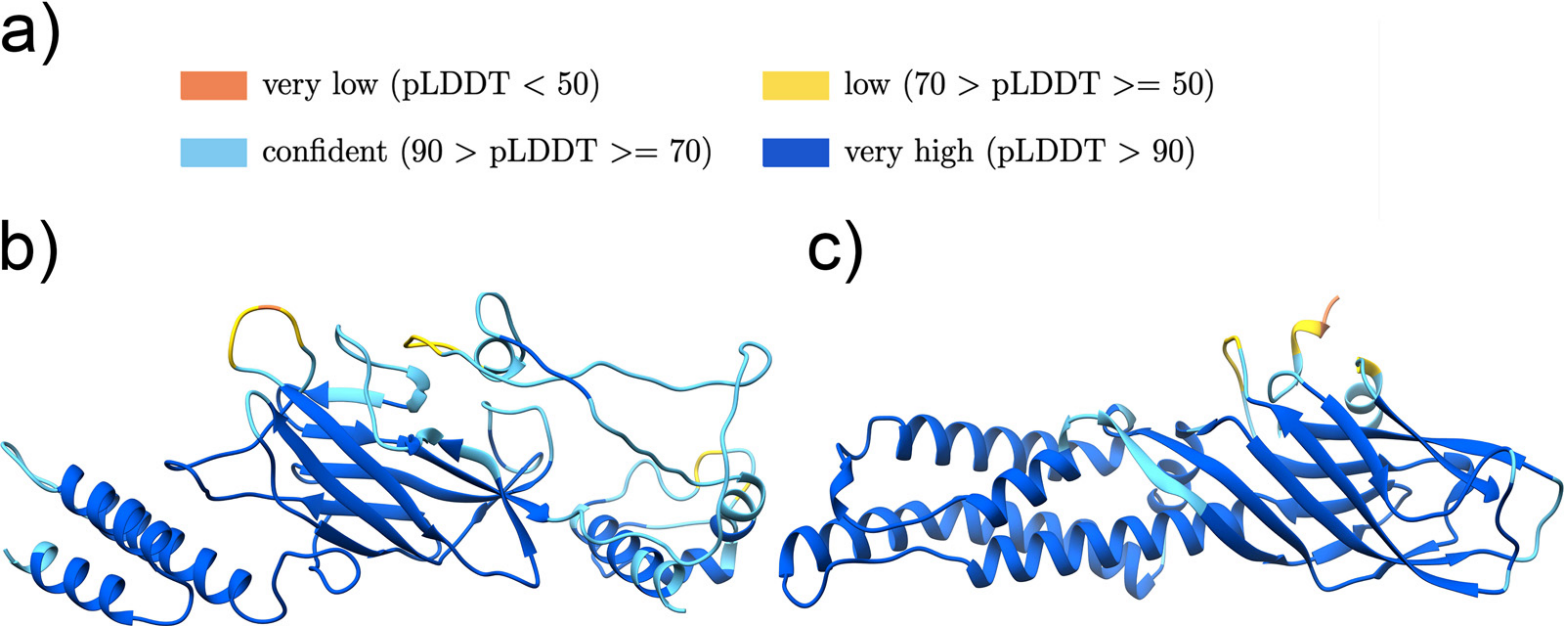
Clusters with AlphaFold structures showing ambiguous adhesion function. (a) Color legend representing the quality of the AlphaFold models. (b and c) Structure models for cluster 3 (UniProtKB accession no. A0A1Q8E8C7 pos. 76-420) (b) and cluster 13 (UniProtKB accession no. A0A069CUH0 pos. 64-357) (c). The figures were produced using Chimera (42).

The best DALI hit for the cluster 3-structure model (Fig. 7b) is an N-terminal helical domain of a group B Streptococcus immunogenic bacterial adhesin named BibA (PDB ID 6POO:A) (Fig. S5b), which superposes with the N-terminal alpha helices of the structure model (31). Running the DALI search for the domain in the middle of the structure model separately results in the S. aureus SdrD adhesive protein (PDB ID 4JDZ:A), where the structure model superposes with the SdrD_B stalk domain (Pfam ID PF17210). Cluster 3 includes one Streptococcus merionis protein (UniProtKB accession no. A0A239SMH4), which is encoded by the *bca* gene. The *bca* gene has been shown to be involved in the initial stage of group B Streptococcus infection (32), suggesting adhesion function.

The best DALI match for cluster 13 is the human integrin alpha-5 protein (PDB ID 7NXD:A), followed by the best bacterial match being the N-termini of the S. gordonii adhesin Sgo0707 (PDB ID 4IGB:B) (Fig. 7b; Fig. S5c). Here, the structure model aligns to the Sgo0707_N2 domain. The cluster includes an E. faecalis protein (UniProtKB accession no. Q82YW8) encoded by the ef3314 gene, which was described to contribute to the virulence properties of this pathogen (33).

**FIG 8:**
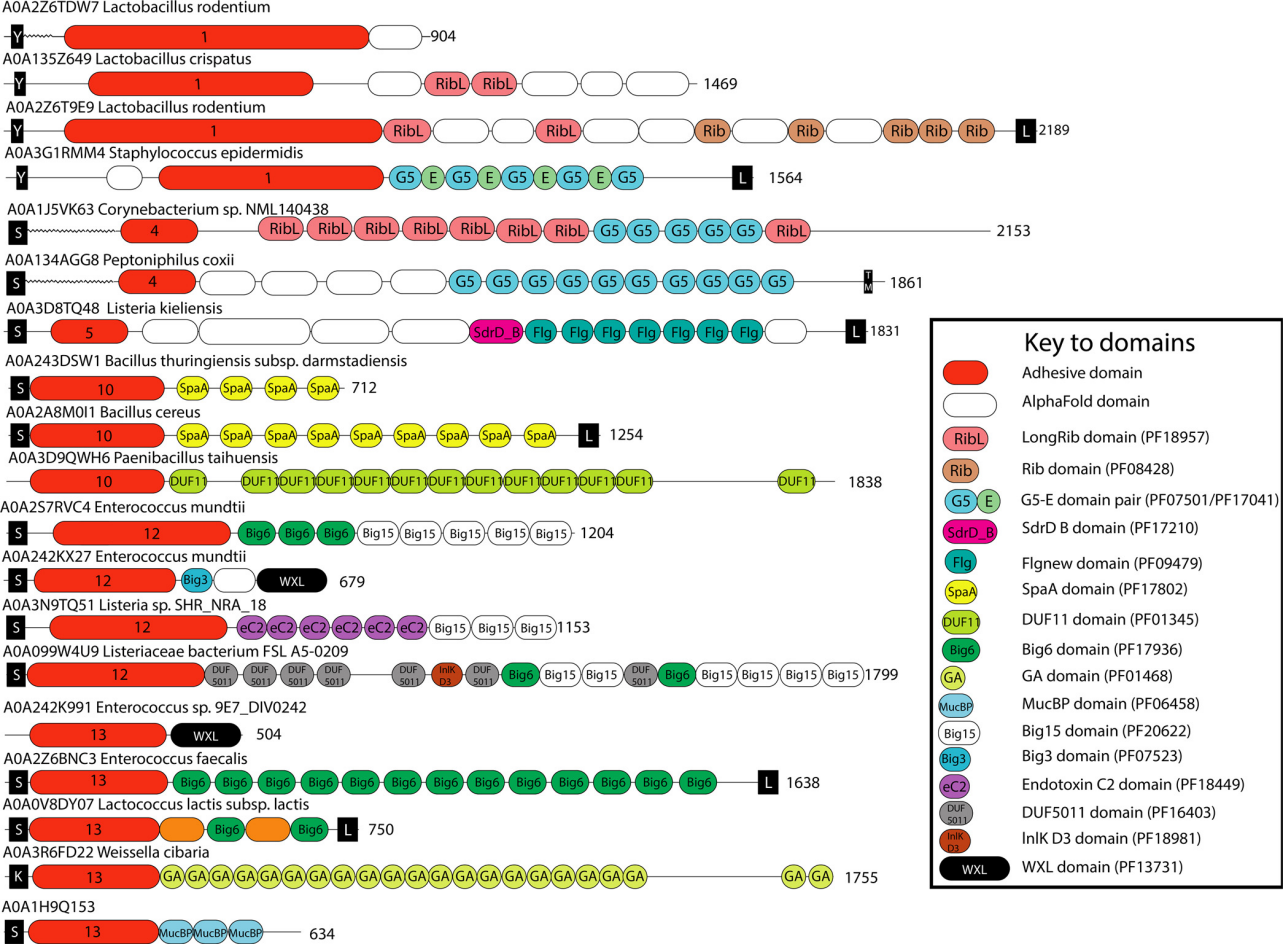
Examples of protein architectures in which the potential novel adhesive domains can be found. The potential novel adhesive domains are annotated in red, labeled with the cluster number. The white domains (“AlphaFold domains”) are domains found in the AlphaFold structure model of each protein, which do not correspond to existing Pfam domain families.

We created new putative adhesive Pfam domain families for clusters 1, 4, 5, 10, 12, 13, and 24. Clusters 1 and 12 were combined into a single cluster. The Pfam identifiers can be found in Table S4. The common domain architectures of proteins with these potential novel members of adhesive domain families are shown in Fig. 8.

### Clusters with stalk-like domains.

**FIG 9:**
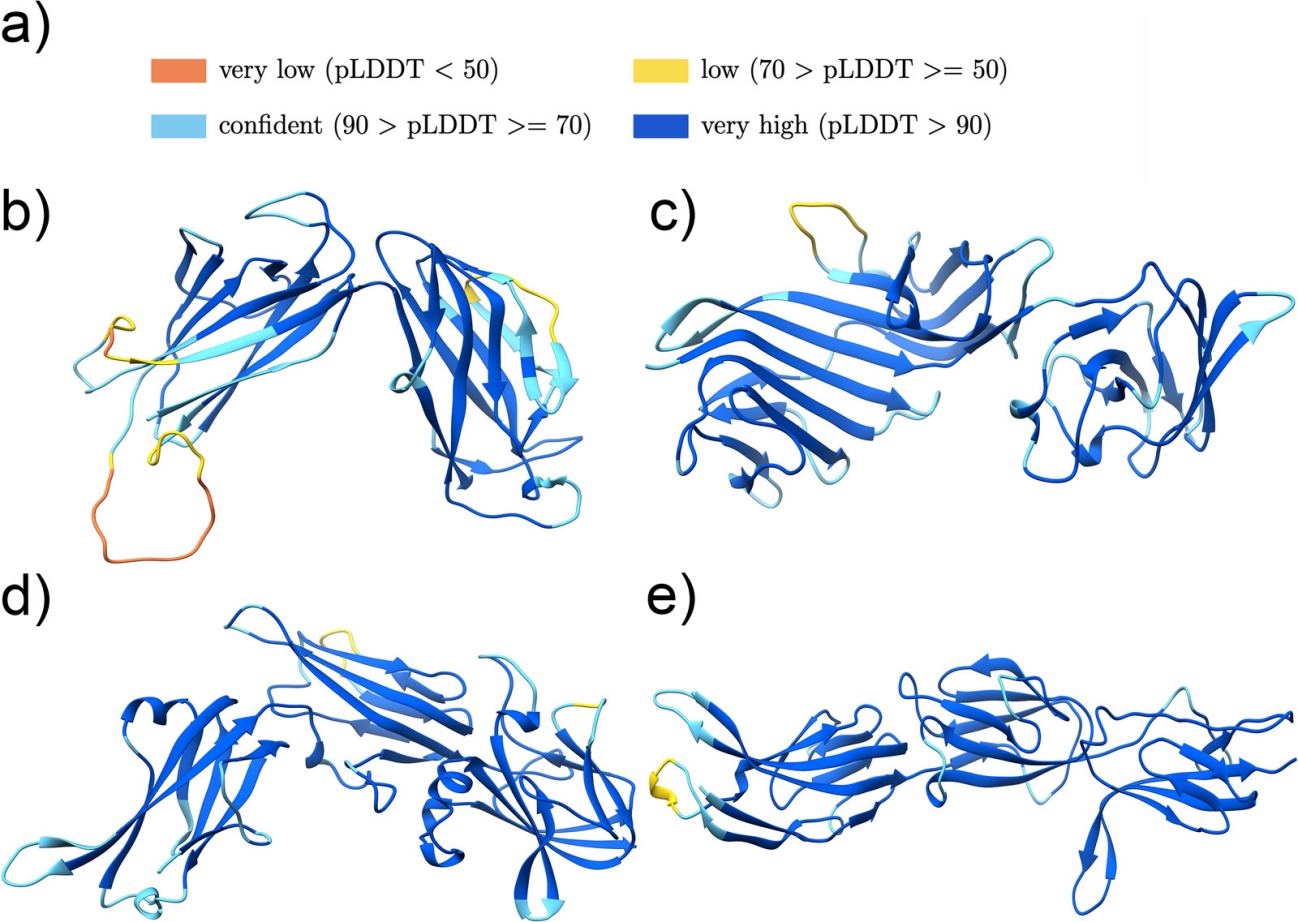
Cluster with AlphaFold structure models with stalk characteristics. (a) Confidence of AlphaFold models color legend. (b to e) Structure models for cluster 11 (UniProtKB accession no. A0A2V5K856 pos. 38-347) (b), cluster 14 (UniProtKB accession no. A8MK03 pos. 34-337) (c), cluster 20 (UniProtKB accession no. A0A1I6IKX0 pos. 39-366) (d), and cluster 22 (UniProtKB accession no. R7HBU9 pos. 38-352) (e).

The Jackhmmer search for clusters 11, 14, 20, and 22 indicated known stalk domains (Table 7). The structure predictions support these results (Fig. 9 and Fig. S6), suggesting the N-terminal region to be composed of stalk domains without a functional N-terminal adhesive domain. As discussed in our previous work, the boundary between adhesive and stalk domains is not always clear, opening the question of whether stalk domains can develop binding functions (7). Additionally, we can find stalk domain structures with similarities to adhesive domains; for example, the DUF11 domain family (Pfam ID PF01345) has similarities to the Collagen_bind adhesive domain structure (Pfam ID PF05737).

**TABLE 7:** Information about stalk-like domain clusters

Cluster no.	Avg. score^*a*^	Cluster size (no. of sequences)	Domain overlap^*b*^	UniProtKB^*c*^	UniProt Reference Proteomes^*c*^	MGnify^*c*^
11	0.82 ± 0.05	10	DUF11 (PF01345)	476^*d*^	87^*d*^	1,237^*d*^
14	0.8 ± 0.04	22	TIG (PF01833)	988^*d*^	207^*d*^	2,190^*d*^
20	0.75 ± 0.03	10	Big_2 (PF02368)	915^*d*^	228^*d*^	9,914^*d*^
22	0.73 ± 0.02	6	Big_2 (PF02368)	109^*d*^	35^*d*^	3,285^*d*^

a Data shown are the average protein prediction score per cluster.

b Similar Pfam adhesive domains found with Jackhmmer are indicated.

c The numbers of homologous sequence hits per cluster in the UniProtKB, UniProt Reference Proteomes, and MGnify databases are shown.

d N-terminal domain.

A second possible function of these proteins is to act as steric regulators altering the access of other adhesive proteins to binding partners. One example is the S. aureus periscope protein SasG, which is suggested to block the binding of proteins located closer to the cell surface from interacting with host cell fibrinogen (9).

The Jackhmmer search for cluster 11 resulted in the known DUF11 (Pfam ID PF01345) stalk domains. The predicted structure shows two distinct domains (Fig. 9b). The DALI search indicated, for the N-terminal domain, a similarity to the stalk-like structure of an integrin alpha-X protein (PDB ID 4NEN:A) (Fig. S6b) and, for the C-terminal domain, a similarity to the BcpA major pilin subunit (PDB ID 3RKP:A).

The sequence, as well as the structure, resembled a TIG stalk domain for the C-terminal domain of cluster 14, indicated by Jackhmmer and the DALI search (PDB ID 5l5G:D) (Fig. 9c; Fig. S6c). The N-terminal part is composed of two subdomains which seem to mirror each other. This symmetry could be based on an internal duplication event. The best DALI hit with a Z-score of 8.1 for the N-terminal part was a plexin-C1 protein (PDB ID 6VXK:D), where the cluster aligns to the two TIG domains in the protein, whereby only the N-terminal subdomain superposes well (Fig. S6c). But separately, both subdomains of the N-terminal part superpose reliably to a TIG domain, suggesting the N-terminal part to be related to a combination of two TIG domains, which might have developed further. A groove on the surface of the structure model indicates that the N-terminal part might have developed a binding function (Fig. 9c).

The structure model for cluster 20 presents three domains (Fig. 9d). The Jackhmmer search already indicated a Big_2 stalk domain, which is supported for the N-terminal domain within the top DALI results (PDB ID 2l04:A) (Fig. S6d). The middle domain resembles a stalk-like structure in Intimin_C (PDB ID 1F00:I), and the best DALI hit for the C-terminal domain was the CfA/I fimbrial subunit A (PDB ID 6K73:B).

The sequence of cluster 22 also indicated a Big_2 stalk domain. The predicted structure again represents three domains, of which the N-terminal and C-terminal domains are most similar to the stalk domains PKD_4 (PDB ID 4U7K:G) and Big_2 (PDB ID 4HU8:C), respectively (Fig. 9e; Fig. S6e). The best DALI hit for the domain in the middle is a monooxygenase (PDB ID 1YEW:E) closely followed by the I-set immunoglobulin-like stalk domain (PDB ID 5AEA:A).

### Clusters with ambiguous function.

The Jackhmmer and DALI search could not indicate explicit binding function for cluster groups 7, 9, 16, 18, and 23 (Table 8; Fig. S7).

**TABLE 8:** Information about clusters of ambiguous function

Cluster no.	Avg. score^*a*^	Cluster size (no. of sequences)	Domain overlap^*b*^	UniProtKB^*c*^	UniProt Reference Proteomes^*c*^	MGnify^*c*^
7	0.84 ± 0.1	8	Beta_helix (PF13229)	6,056	1,463	66,262
9	0.83 ± 0.07	7	PSII_BNR (PF14870)	15,047	2,460	86,845
16	0.78 ± 0.08	11	BNR_4 (PF15892)	7,916	1,148	37,575
18	0.77 ± 0.09	11	Peptidase_M26_N (PF05342)	10,570	2,857	350,932
23	0.71 ± 0.06	17	DUF3344 (PF11824)	3,408	755	9,724

a Data represent average prediction score of the proteins per cluster.

b Data show information regarding overlapping known Pfam domain families found with Jackhmmer.

c The numbers of homologous sequence hits per cluster in the UniProtKB, UniProt Reference Proteomes, and MGnify databases are shown.

The Jackhmmer search indicated a Beta_helix (Pfam ID PF13229) for cluster 7 and a Peptidase_M26_N (Pfam ID PF05342) domain for cluster 18. The structure models of both domains resemble carbohydrate binding pectate lyase adhesive domains. But the top DALI hit for cluster 7 is a lacto-*N*-biosidase (PDB ID 6KQS:A) and, for cluster 18, a putative immunoglobulin protease (PDB ID 3N6Z:A), suggesting potential catalytic functions (34).

Jackhmmer indicated a BNR_4 (Pfam ID PF15892) domain for cluster 16, and the top DALI hit for this cluster is a human integrin alpha-IIb protein, which binds, among others, to fibrinogen. The InterPro database has a Fucose_binding_lectin domain annotated (PDB ID 1IUB), suggesting cluster 16 as having adhesion function (35, 36). The structure model of cluster 9 resembles cluster 16, whereby the top DALI hit is a virginiamycin B lyase (PFB ID 2Z2O:B), again suggesting catalytic function (37).

The best DALI hit for cluster 23 was the P_proprotein domain (Pfam ID PF01483) of a protease, which is common to be located downstream of a catalytic domain (PDB ID 3HJR:A) (38).

**FIG 10:**
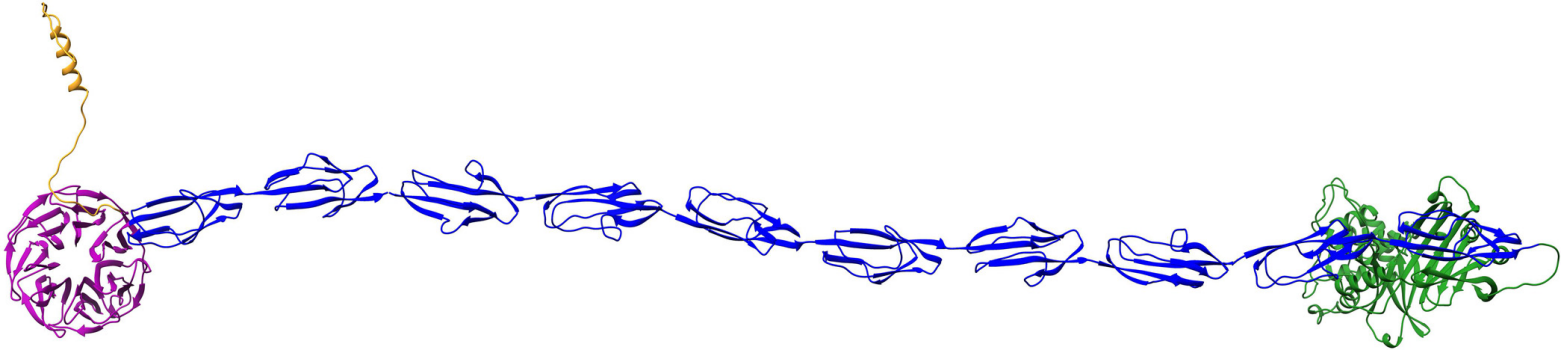
Example and predicted structure of a potential fibrillar enzyme (UniProtKB accession no. C6CUY3). The structure was predicted using the AlphaFold colab notebook, where three sequence chunks (residues 1 to 600, 401 to 1,000, and 901 to 1,500) were predicted separately with overlapping regions, which were combined using PyMOL (45). The potential sorting signal region is colored in yellow, the potential catalytic domain in violet, the Flg_new stalk domains in blue, and the anchor region in green. This protein belongs to sequence cluster 9. This figure was produced using Chimera (42).

The results of the above-described clusters suggest the clusters play a catalytic role, not ruling out binding abilities. Given that catalytic domains often also have adhesive function to bind to their substrate, it is challenging to differentiate between catalytic and adhesive domains and also between fibrillar adhesins and, as we call them, “fibrillar enzymes.” Fibrillar enzymes are also composed of repeating domains but have an enzymatic-related domain instead of an explicit adhesive domain (Fig. 10). Nevertheless, the enzymatic region can be able to have binding functions, as described above.

## DISCUSSION

Novel pathogens are emerging constantly with uncharacterized host-cell interaction mechanisms. Homologous virulence-associated proteins with known adhesive domains are the first step toward an understanding of the pathogenicity of these bacteria. But adhesive domains evolve quickly and are highly variable. Hence, detection mechanisms independent of known adhesive domains are important. In this study, we developed a random forest-based discovery approach to detect FA-like proteins. We applied the approach to the *Firmicutes* and *Actinobacteria* UniProt Reference Proteomes database, yielding over 6,500 confidently predicted FA-like proteins.

With the characterization of FA-like proteins, we could identify a variety of notable features which we could use for machine learning. The known stalk and adhesive domains are the strongest feature in the classification decision approach. This bias is due to the positive training data, which were selected from the prior domain-based discovery approach (7). Other strong features were the protein length, which is required to overcome the bacterial cell surface, and the amino acid composition of the protein sequences. Here, particularly threonine was strongly overrepresented in the positive training data set compared to the negative training data set, raising the question of what role it plays in bacterium-host interactions. Fibrillar adhesins are cell surface proteins, and so, we selected the existence of a cell wall anchor motif or domain as an additional feature, although an anchor was found in only around half of the proteins of the positive training data. One reason could be that there are many unknown anchor motifs or domains, which still need to be investigated, or nonclassical secretion mechanisms (39). We also found many examples of potential fibrillar adhesins where the stalk region ranges to the C-terminus. These proteins might be able to interact with other cell surface proteins in order to be projected away from the cell wall. Implementing the selected identification properties in the random forest classification approach and applying it to the *Firmicutes* and *Actinobacteria* UniProt Reference Proteomes database led to over 6,500 confidently detected FA-like proteins. This indicates that more than 5,000 of them were missed by the domain-based discovery approach detected in our previous study (7). More importantly, with our new machine learning discovery approach, FA-like proteins are predicted that lack known adhesive or stalk domains, enabling us to discover novel protein domain families.

To verify the random forest prediction approach, we further studied the predicted FA-like proteins lacking a known adhesive domain, but with known stalk domains. When investigating the sequence clusters representing an annotation gap N-terminal to known stalk domains, similar Pfam domain sequence matches could be found for many of the described clusters by using Jackhmmer. This suggests that the Pfam domain families could be expanded to include these sequences, or novel domain families related to the overlapping domain families can be created. We have taken advantage of the recent release of the AlphaFold2 software to validate our machine learning approach, as well as use it to refine predictions of adhesive domains in our predicted fibrillar adhesins. Given that the predicted structures confirm the Jackhmmer results, this highlights the high accuracy of the structure prediction method AlphaFold2. We see many new opportunities to use large-scale structure predictions to identify and investigate the components of the bacterial cell surface that are likely to interact with the host.

While further investigating the described N-terminal sequence clusters, the difficulty in differentiating between fibrillar adhesins and the newly discovered class of fibrillar enzymes was shown. Given that fibrillar enzymes can play an important role in bacterial pathogenesis as well, they have comparable characteristics to fibrillar adhesins in terms of being long surface proteins with a stalk and that several of the enzymes can have binding functions. This impedes the differentiation of these two protein classes by our identification features, and so far, we have not included any property to differentiate between adhesive and enzymatic domains. Nevertheless, the prediction score and the cluster size can together give an assessment of the reliability. The analyzed clusters with the higher sequence number or higher prediction score are mostly potential adhesive domains.

Except for clusters 3 and 13, the sequences or predicted structures of the other potential adhesive domain clusters are similar to known adhesive domains. Additionally, the potential novel adhesive domains, as well as the clusters related to known adhesive domains found with Jackhmmer, verify the random forest-based discovery approach. The high number of homologous sequences of these domains in the metagenomic MGnify database and known pathogenic genera in the UniProt database underline their relevance. For the potential novel members of adhesive domain families discovered in the course of this study, the predicted structure models and the DALI search results give a first understanding of their function and potential binding partners. AlphaFold2 and AlphaFold-Multimer open up further ways to predict the structures of fibrillar adhesins-target protein complexes (40). We believe that we are at the beginning of a new age of discovery where computational analyses will lead to fundamental improvements in our understanding of microbial-host interactions.

## MATERIALS AND METHODS

### Training data selection.

We selected, as positive training data, the FA-like proteins of *Actinobacteria* and *Firmicutes* discovered with the domain-based detection approach in our previous study (7). Additionally, we included 25 additional FA-like proteins that do not have a known adhesive and stalk domain, which were found in the literature or manually investigated. As negative training data, we randomly selected non-FA-like proteins in Reference Proteomes database in which FA-like proteins could be detected with the domain-based discovery approach. These are from the following nine organisms: Bifidobacterium subtile, *Olsenella* species oral, Slackia exigua, Streptomyces coelicolor, Staphylococcus aureus, Lactococcus lactis, Streptococcus gordonii, Listeria monocytogenes, and Enterococcus faecalis. The training set consists of a total of 3,332 proteins, of which half belong to the positive and the other half to the negative training data set. The training data can be found in the GitHub repository (see below).

### Identification features calculation and random forest classification.

To search in the protein sequences for known adhesive, stalk, and anchor domains, the collection of Pfam domain HMMs from our previous study was used (7). Additionally, the adhesive domain GspA_SrpA_N (Pfam ID PF20164) and the stalk domains aRib (Pfam ID PF18938), RibLong (Pfam ID PF18957), SasG_E (Pfam ID PF17041), GA-like (Pfam ID PF17573), YDG (Pfam ID PF18657), Lipoprotein_17 (Pfam ID PF04200), and IgG_binding_B (Pfam ID PF01378) were used. These HMMs were run against the protein sequences using the HMMER tool (version 3.1b2) with the gathering (GA) threshold option. Using regular expression, we searched within the C-terminal 50 residues of the protein sequences for the following sortase anchor motifs: LPxTG, LPxTA, LPxTN, LPxTD, LPxGA, LAxTG, IPxTG, NPxTG, and NPQTM (“x” can be any amino acid).

To identify highly similar tandem sequence repeats that may represent potential unknown stalk domains, we applied the T-REKS software to the sequences, using, as parameters, a minimum of 70% sequence identity, 50 residues as minimum length of the repeat region, and 5 residues as minimum seed length (12).

Disordered regions were predicted using IUPred (IUPred2a) with the IUPred2-type “long” for predicting long disordered regions (13). Each residue with an IUPred score above 0.5 was counted as predicted disordered. The predicted disordered fraction was calculated using the percentage of predicted disordered residues from the total protein length.

The proportion of charged or hydrophobic amino acids per protein sequence was calculated using, as charged amino acids, glutamic acid (E), aspartic acid (D), lysine (K), and arginine (R) and, as hydrophobic amino acids, alanine (A), isoleucine (I), leucine (L), methionine (M), phenylalanine (F), tryptophan (W), tyrosine (Y), and valine (V).

The residue length was counted per the complete UniProt protein sequence.

The proportion for each amino acid per protein sequence was calculated, and to evaluate the amino acid composition bias, the relative entropy (Kullback-Leibler [KL] divergence) was calculated per protein sequence (*S*). Here, we quantify the difference between the observed frequency (*P*) per amino acid (*i*) compared to equally frequent amino acids, being 0.05 for 20 amino acids.

$$\text{KL}(S)=\sum_{i}^{20}P(i)\text{log}\frac{P(i)}{0.05}$$

We calculated the identification features for the protein sequences of the training data. With the calculated feature data, we trained a random forest classifier from sklearn.ensemble methods with 50 trees with a maximum of 3 features per tree and random state 2 (41). The random forest method takes the 30 features as input and outputs a score per protein between 0 and 1, with FA-like proteins scoring closer to 1.

The reliability curve was calculated for the applied random forest model on the training data set using calibration_curve from the sklearn calibration module and a 10-fold cross-validation approach.

For calculating the precision and recall of the model and generating the precision recall curve, we generated a testing data set of 258 proteins, composed of 128 FA-like proteins and 130 non-FA-like proteins. We artificially adapted the features of the testing set to have no adhesive or stalk domains, whereby all other features were retained. For these calculations, the proteins of the testing set were excluded from the training data set. The precision and recall of the model, as well as the precision recall curve, were calculated using the macroaverage method to determine how the random forest model performs overall across the two classes, FA-like and non-FA-like proteins. The precision recall curve was also calculated using a cross-validation approach with the training set.

To use the random forest discovery approach, we provide the code in our GitHub repository (see below).

### FA-like proteins prediction for *Firmicutes* and *Actinobacteria* UniProt reference proteomes.

To apply our machine learning method against known *Firmicutes* and *Actinobacteria* proteins, we first gathered available sequences. The UniProt proteome identifier for all *Firmicutes* and *Actinobacteria* reference proteomes was searched for on the UniProt website (release 2020_04). We collected the relevant sequences for these identifiers by searching in the knowledgebase under the bacterial reference proteomes (release 2020_03) for the identifier.

As described in the subsection “Identification features calculation and random forest classification,” we calculated the identification features for the *Firmicutes* and *Actinobacteria* reference protein sequences and applied the trained random forest classification approach to score each protein.

### Analyzing predicted FA-like proteins.

We further analyzed the predicted FA-like proteins by differentiating the prediction scores. The subcellular localizations of the predicted FA-like proteins were predicted using singularity (version 3.5.3) to run the PSORTb (version 3) Docker image (psortb_commandline_1.0.2.sif).

To find distantly related adhesive domains, a profile HMM search with the known adhesive domains was conducted using an E-value threshold of 1.0.

Using profile HMM-search (version 3.1b2) with the GA threshold option, the Pfam database (version 33.1) was run against the sequences of the predicted FA-like proteins.

### Selecting potential functional sequences.

To verify the machine learning approach, we were particularly interested in the predicted proteins with an annotation gap at the N-terminus, which might contain a missing functional domain. We focused on the N-terminus because we showed in our previous study that the adhesive domain in FA-like proteins in *Firmicutes* and *Actinobacteria* is mostly found at the N-terminus (7). We selected proteins with at least four known stalk domains which lack a known adhesive domain and with no Pfam domain annotations within the first 20% of the protein length. Before finding homologous sequence groups, we deleted the selected protein’s first 20 residues to avoid clustering based on a potential signal peptide. We cut these sequences N-terminal to the first domain annotation but no longer than 400 residues in order to try to avoid clustering based on potential stalk domains. We clustered those excised sequences into homologous sequence cluster groups using BLASTp all against all with an E-value threshold of 0.001, requiring a coverage threshold of 85% and an identity threshold of 25% (16). For each cluster, we calculated the reliability by averaging the random forest prediction scores of the proteins per cluster. We sorted the resulting clusters by average prediction score as well as sequences per cluster. We further investigated the 24 cluster groups with at least 5 homologous sequences.

To investigate the potential function of these sequence clusters, we chose one representative protein per cluster. To do so, we aligned the N-terminal sequences per cluster and manually selected one representative sequence per cluster, which was used for the following investigations.

For each representative sequence, we searched the whole UniProtKB with Jackhmmer by using the HMMER website (https://www.ebi.ac.uk/Tools/hmmer/search/jackhmmer) to find domain families related to the sequence clusters (17). For cluster 5, we found a distant related stalk domain overlapping with the C-terminus of the representative sequences; we trimmed off the sequence with the domain annotation and continued with the N-terminal sequence.

The structure for each representative sequence was predicted with AlphaFold2 using the Google colab repository provided by DeepMind (https://colab.research.google.com/github/deepmind/alphafold/blob/main/notebooks/AlphaFold.ipynb) (11). Based on the predicted structure, we selected the domain boundaries and cut the structure as well as sequence of each cluster accordingly (see Table S2 in the supplemental material). In most cases, we cut off disordered regions. In single cases, for cluster 4, we optimized the structure by cutting off a stalk domain-like C-terminus to focus on the potential adhesive domain and rerun the AlphaFold structure prediction. For clusters 1 and 17, we extended the sequence to include the whole domain.

To assess the quality of the models, AlphaFold stores the pLDDT confidence in the B-factor field of the output PDB files, which were used to color the structure models by quality using Chimera (42). To find out more about the function of the clusters, we searched with the predicted structure models, optimized to the domain boundaries, for similar structures in the PDB database using DALI (43).

We created an HMM from the sequences per cluster based on the detected domains using hmmbuild (17). With these HMMs, we searched against the metagenomic MGnify (release 2019_05), UniProt Reference Proteomes, and UniProtKB (release 2021_01) databases for homologous sequences using a domain E-value threshold of 0.01 (19, 20). From the UniProt website, the Retrieve ID/Mapping tool was used to compare the organisms' information to the UniProtKB matches.

### Data availability.

The AlphaFold structure model, as well as the random forest prediction results, for the *Firmicutes* and *Actinobacteria* reference proteomes can be found in an institutional repository of the University of Cambridge (https://www.repository.cam.ac.uk/handle/1810/335004) (44). We provide a GitHub repository (https://github.com/VivianMonzon/FAL_prediction), which includes the training data set and the code to run the random forest-based FA-like protein prediction on a sequence of interest, as well as a colab notebook (https://colab.research.google.com/github/VivianMonzon/FAL_prediction/blob/main/Colab/ML_FA_prediction.ipynb).
