# Supplementary material for: Large-Scale Discovery of Microbial Fibrillar Adhesins and Identification of Novel Members of Adhesive Domain Families
Source: J Bacteriol. 2022 May 24;204(6):e00107-22. doi: 10.1128/jb.00107-22 (PMC9210967; doi:10.1128/jb.00107-22)
Supplement: Supplemental file 1 — Fig. S1-S7; Tables S1-S4. Download jb.00107-22-s0001.pdf, PDF file, 6.2 MB [file jb.00107-22-s0001.pdf]

Supplementary

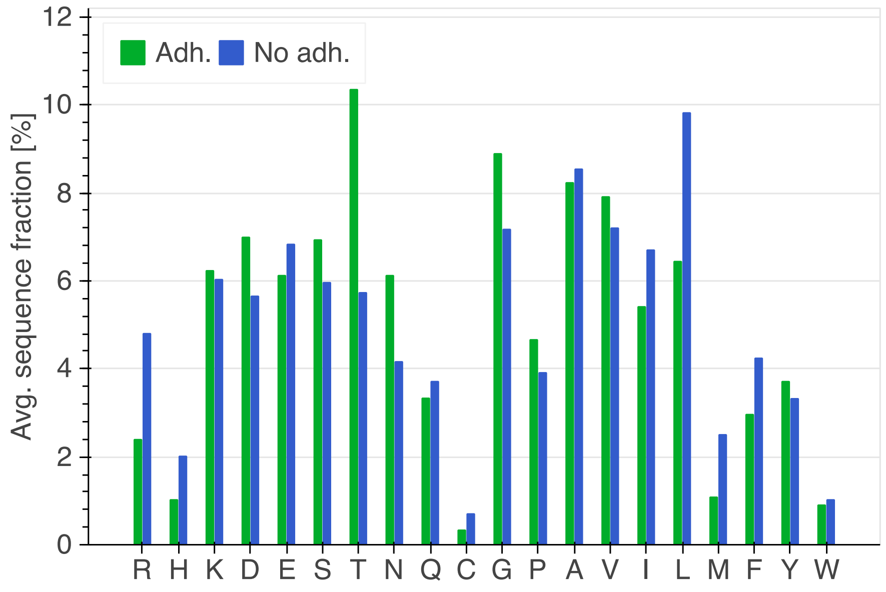

Figure S1: Average amino acid fraction: This bar plot shows for each amino acid the average fraction calculated per positive (Adh.) and negative (No adh.) training data set.

Table S1: FAL per proteome counts: These tables display the information to the reference proteomes with the highest fraction of predicted FA-like proteins with a score of 0.7 or above per reference proteome size for a) Firmicutes and b) Actinobacteria.

a) Firmicutes

**Organism**	**Proteome ID**	**# FA-like proteins**	**Proteome size**	**Percentage**
Listeria booriae	UP000029844	57	3104	1.84
Culicoidibacter larvae	UP000306912	43	2363	1.82
Granulicatella elegans ATCC 700633	UP000002939	20	1562	1.28
Weissella cryptocerci	UP000292886	33	2588	1.28
Listeria monocytogenes serovar 1/2a	UP000000817	36	2844	1.27
Veillonella sp. DORA_B_18_19_23	UP000053161	11	870	1.26
Lactobacillus rodentium	UP000257317	18	1434	1.26

Granulicatella balaenopterae	UP000198556	24	1916	1.25
Clostridium sp. CAG:433	UP000018401	16	1308	1.22
Lactobacillus sp. LL6	UP000319179	22	1826	1.20

b) Actinobacteria

**Organism**	**Proteome ID**	**# FA-like proteins**	**Proteome size**	**Percentage**
Alloscardovia macacae	UP000243657	18	1550	1.16
Rarobacter faecitabidus	UP000315389	25	2325	1.08
Pseudoscardovia suis	UP000216454	18	1732	1.04
Rarobacter incanus	UP000316181	20	2018	0.99
Corynebacterium kutscheri	UP000033457	20	2047	0.98
Micrococcales bacterium KH10	UP000198369	22	2348	0.94
Bifidobacterium primatium	UP000229095	19	2029	0.94
Bifidobacterium indicum LMG 11587	UP000028569	12	1350	0.89
Atopobium vaginae DSM 15829	UP000005947	11	1243	0.88
Bifidobacterium bifidum BGN4	UP000006173	16	1834	0.87

Table S2: Representative sequences: this table lists the representative sequences and the regarding domain positions based on the AlphaFold structure prediction for the N-terminal annotation gaps of predicted FA-like proteins with minimum 4 stalk domains, lacking a known adhesive domain. The DALI search was conducted with the sequence regions listed here.

Nr.	UniProt identifier	Domain info	Start position	End position
1	A0A2Z6T9E9		185	718
2	K8EVB1	N-term	117	256
		C-term	257	417
3	A0A1Q8E8C7		76	420

4	B0S3M8		153	316
5	A0A5R8Q9T8		68	240
6	V2XMF4	N-term	80	239
		C-term	240	366
7	R6V4J4		37	420
8	R3TX93		40	276
9	C6CUY3		40	363
10	A0A0F7RLJ7		46	326
11	A0A2V5K856	Nterm	38	201
		Cterm	201	347
12	A0A099WCN8		48	417
13	A0A069CUH0		64	357
14	A8MK03	Nterm	24	225
		Cterm	225	337
15	A0A4U7JL97		37	420
16	A0A494X6E7		38	377
17	A0A2Z4U801		240	508
18	R5FG57		42	394
19	S0JHJ4		32	199
20	A0A1I6IKX0	Nterm	39	142
		Middle	143	244
		Cterm	244	366
21	A0A373LEL7	Nterm	30	196
		Cterm	197	420
22	R7HBU9	Nterm	38	158
		Middle	158	251
		Cterm	252	352
23	Q81AL6		21	339
24	B1BZ86		36	324

Table S3: Structure model DALI results info: The table lists the top DALI hits of the AlphaFold models for the N-terminal sequence clusters with the regarding Z-scores and similarity information when the structures are superposed using pymol. The Notes column gives information about the similarities to structures alternative to the top DALI hit, which were described in the main text.

Nr.	Domain	Top DALI hit	Z-score	Superposition (RMSD)	Notes
1		5ny0:A	27.3	5.07 Å over 272 res.	
2	N-term	4b60:B	11.5	3.42 Å over 128 res.	
	C-term	4igb:B	13.7	3.27 Å over 128 res.	4jdz:A -> 3.74 Å over 136 res.
3		6poo:A	10.5	3.24 Å over 64 res.	4jdz:A -> 3.89 Å over 104 res.
4		2x9x:A	7.5	4.43 Å over 112 res.	
5		5xcb:A	10.8	4.52 Å over 128 res.	
6	N-term	5cf3:A	11.1	4.57 Å over 128 res.	
	C-term	2z1p:A	11.7	3.50 Å over 120 res.	
7		6kqs:A	52.8	1.42 Å over 352 res.	
8		6fx6:A	20.8	3.78 Å over 224 res.	
9		2z2o:B	31.1	3.04 Å over 272 res.	
10		4nuh:A	15.2	4.30 Å over 176 res.	
11	Nterm	4nen:A	11.5	4.06 Å over 120 res.	
	Cterm	3rkp:A	12.4	3.35 Å over 128 res.	
12		5ny0:A	31.1	3.39 Å over 280 res.	
13		7nxd:A	10.1	6.96 Å over 72 res.	4igb:B -> 4.88 Å over 72 res.
14	Nterm	6vxk:D	8.1	7.37 Å over 120 res.	
	Cterm	5l5g:D	9.7	3.99 Å over 88 res.	
15		3bz5:A	37.7	3.00 Å over 352 res.	
16		6v4p:A	28.9	4.64 Å over 272 res.	
17		6fwv:B	13.6	7.61 Å over 224 res.	
18		3n6z:A	32.6	4.68 Å over 288 res.	
19		6fx6:A	14.6	4.19 Å over 128 res.	5A0L:B -> 4.26 Å over 144 res.
20	Nterm	4uid:B	9.1	3.94 Å over 72 res.	2l04:A -> 2.92 Å over 64 res.

	Middle	1f00:I	8.7	4.19 Å over 80 res.	
	Cterm	6k73:B	6.8	5.48 Å over 80 res.	
21	Nterm	6fx6:A	11.7	4.22 Å over 120 res.	
	Cterm	3kpt:A	9.2	6.11 Å over 80 res.	
22	Nterm	4u7k:G	8.9	3.46 Å over 80 res.	
	Middle	1yew:E	7.6	4.24 Å over 80 res.	5aea:A -> 3.69 Å over 88 res.
	Cterm	4hu8:C	8.8	3.99 Å over 72 res.	
23		3hjr:A	8.7	4.46 Å over 128 res.	
24		6fwy:B	13.4	5.35 Å over 232 res.	

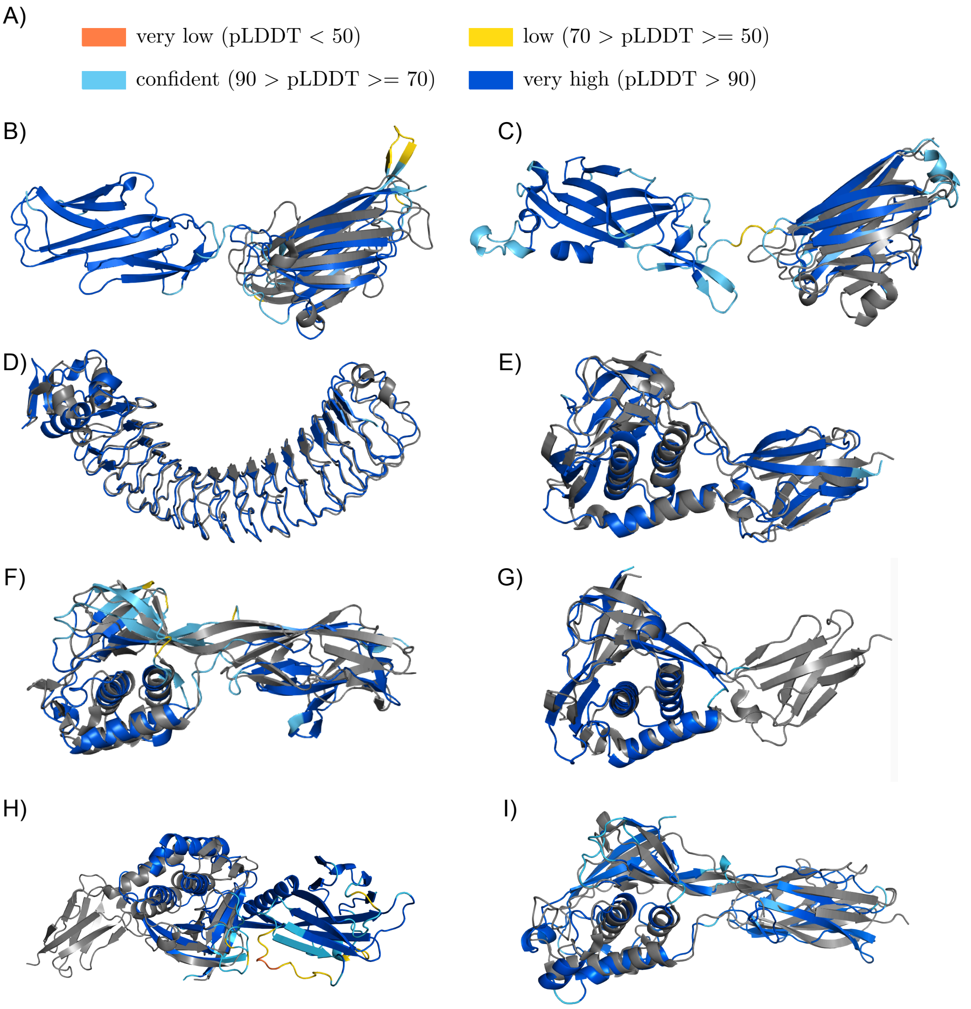

Figure S2: Superposition of structure models shown in figure 5 with sequence similar adhesive domains found by Jackhmmer: a) Colour legend representing the quality of the AlphaFold models. b) Superposition of the structure model of cluster group 2 with the SdrG_C_C binding domain (PDB:4jdz:A). c) Superposition of Collagen_bind domain (PDB:2z1p:A) to the C-terminal domain of the cluster group 6 structure model. d) Predicted structure model for cluster group 15 superposed with the functional region of Internalin J (PDB:3bz5:A). e) Predicted structure model for cluster group 8 superposed with S. aureus TED adhesive domain (PDB:6fx6:A). f) Predicted structure model of group 17 superposed with the Bacillus anthracis TED adhesive domain (PDB:6fwv:B). Superposition of S. aureus TED domain (PDB: 6fx6:A) and the structure model for g) cluster 19 and h) cluster 21. i) Cluster 24 structure model superposed to thioester domain of E. faecium (PDB: 6fwy-B).

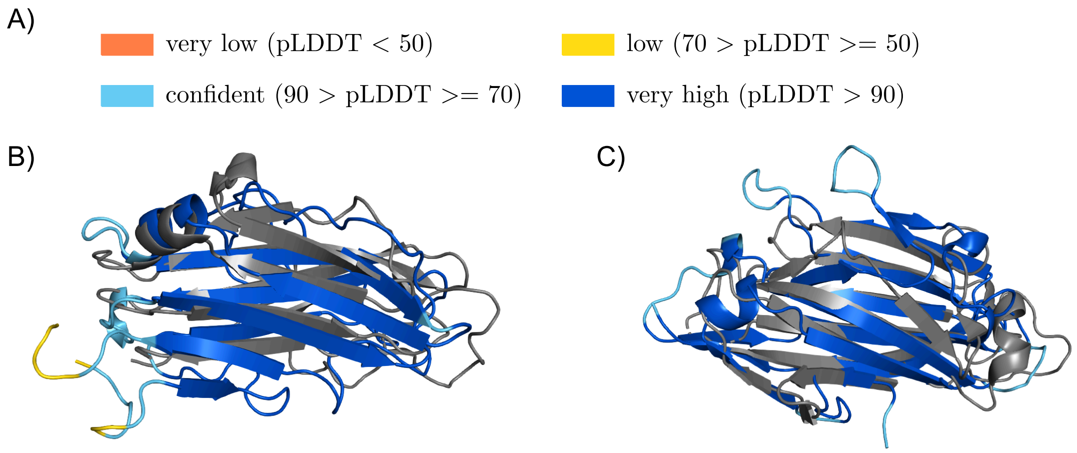

Figure S3: Superposition with the DALI detected PDB structures similar to the clusters showing a jelly-roll like structure. a) AlphaFold colour legend for the structure prediction confidence. b) Superposition of the structure model for cluster group 4 with the GramPos_pilinBB domain (PDB:2x9x:A). c) Predicted structure model for cluster group 5 superposed with the GramPos_pilinBB domain (PDB:5xcb:A).

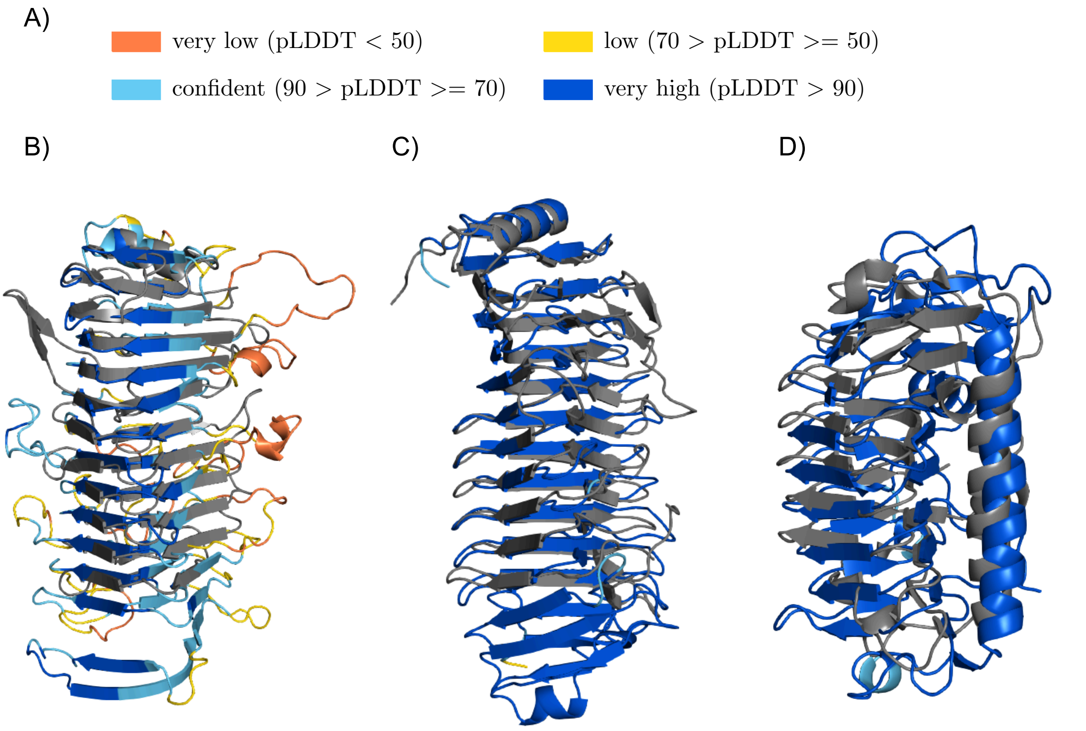

Figure S4: Beta-solenoid predicted structure superposed with the top DALI matches in the PDB database. a) AlphaFold prediction confidence colour legend. Superposition with the adhesive region of the serine-rich Lactobacillus reuteri adhesin (PDB:5ny0:A) with the b) predicted structure model for cluster group 1 and c) predicted structure for cluster group 12. d) Superposition of the structure model for cluster group 10 with the Ice_binding domain (PDB:4nuh:A).

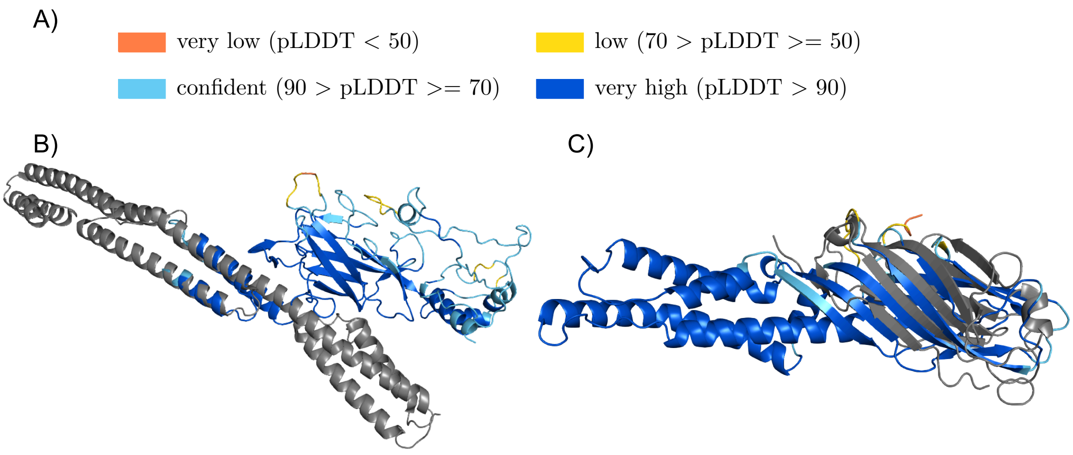

Figure S5: Superposition of clusters with ambiguous adhesion function with DALI hits: a) Confidence colour legend for AlphaFold predicted structures b) Cluster 3 structure model superposed with the immunogenic adhesin BibA (PDB:6poo:A). c) Cluster 13 structure model superposed with the S. gordonii Sgo0707_N2 domain (PDB:4igb:B).

Table S4: Pfam families created based on this study

Cluster	Pfam accession	Pfam identifier
1+12	PF20585	Pectate_lyase_5
4	PF20592	pAdhesive_10
5	PF20595	pAdhesive_6
10	PF20597	pAdhesive_15
13	PF20609	pAdhesive_17
24	PF20610	TED_2

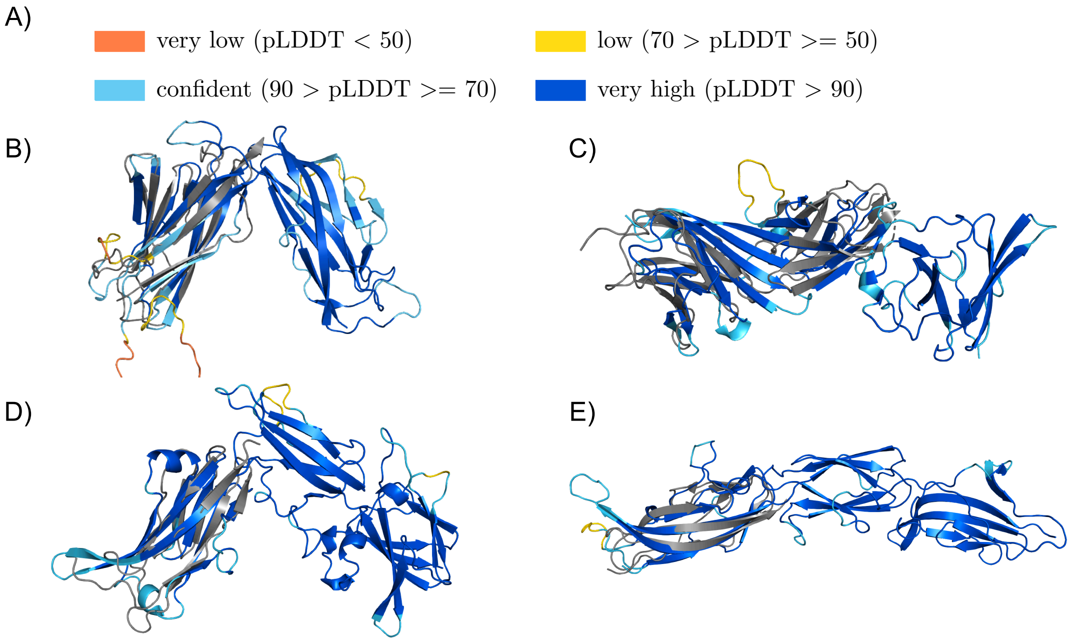

Figure S6: Superposition of clusters with potential stalk characteristics with DALI hits: a) Colour legend of AlphaFold prediction confidence b) Cluster 11 structure model superposed to stalk of an Integrin alpha-X protein (PDB: 4nen:A). c) Superposition of prediction structure for cluster 14 with the TIG domains of the plexin-C1 protein (PDB: 6vxk:D). d) Superposition of the N-terminal domain of the prediction structure for cluster 20 with Big_2 stalk domain of the major tail protein V (PDB: 2l04-A). e) Cluster 22 structure model superposed to the PKD_4 stalk domain of a collagenase ColH (PDB: 4u7k:G).

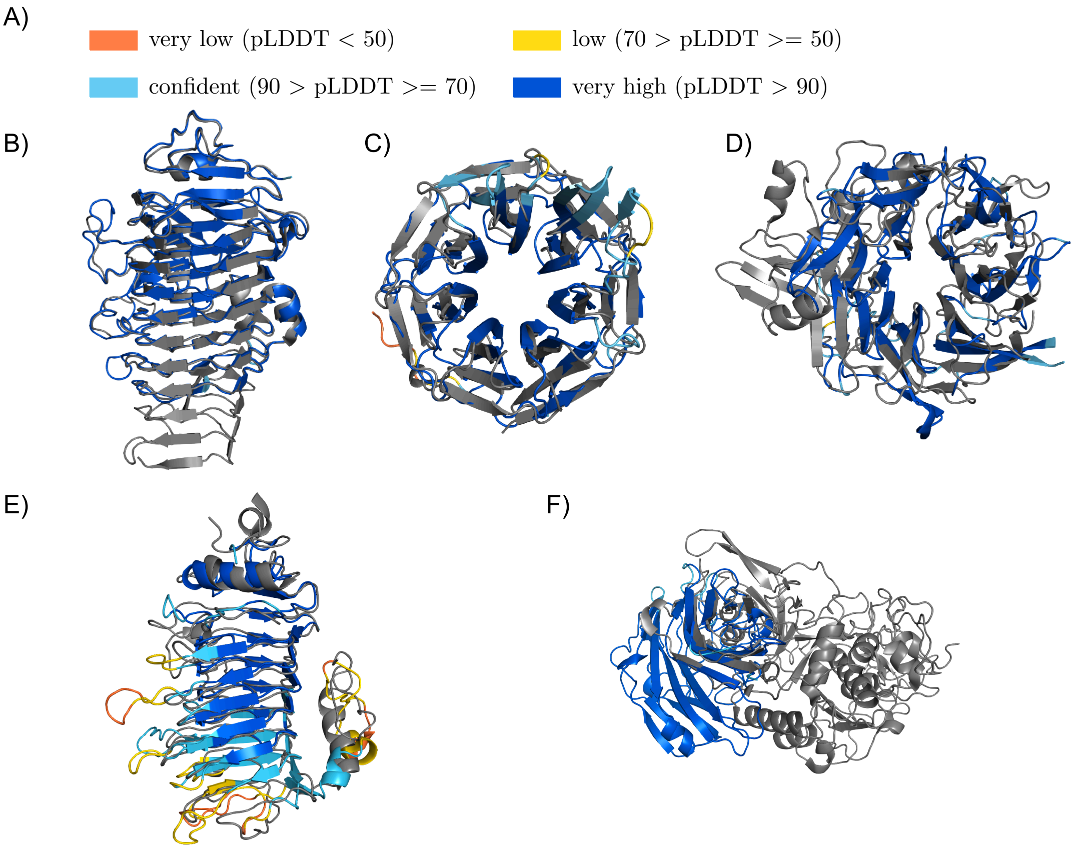

Figure S7: Predicted structures for clusters with unlikely adhesion function: (a) Colour legend for AlphaFold models quality. (b) Cluster 7 structure model superposed with an Eubacterium lacto-N-biosidase (PDB:6kqs:A). (c) Superposition of cluster 9 structure model with a virginiamycin B lyase (PDB:2z2o:B). (d) Cluster 16 structure model aligned with an human integrin alpha-IIb/beta-3, which binds among others to fibrinogen (PDB: 6v4p:A). (e) Superposition of cluster 18 structure model with a putative immunoglobulin protease (PDB: 3n6z:A), whereby the structure strongly resembles a pectate lyase. (f) Structure model of cluster 23 superposed to the P_proprotein domain of a protease (PDB: 3hjr:A).
